# The general equation of δ direct methods and the novel *SMAR* algorithm residuals using the absolute value of ρ and the zero conversion of negative ripples

**DOI:** 10.1107/S2053273324009628

**Published:** 2025-01-01

**Authors:** Jordi Rius

**Affiliations:** a Institut de Ciència de Materials de Barcelona (CSIC) Campus de la UAB 08193Bellaterra Catalonia Spain; Helmholtz Centre for Infection Research, Germany

**Keywords:** *SMAR* phasing algorithm, δ-*GEQ*, δ direct methods, *SMAR* residuals, *ipp* density modification, crystal structure solution, origin-free modulus sum function, δ recycling algorithm, δ_M_ tangent formula

## Abstract

The general equation δ_*M*_(**r**) = ρ(**r**) + *g*(**r**) of the δ direct methods (δ-*GEQ*) is established which, when expressed in the form δ_*M*_(**r**) − ρ(**r**) = *g*(**r**), is used in the *SMAR* phasing algorithm [Rius (2020). *Acta Cryst* A**76**, 489–493]. It is shown that *SMAR* is based on the alternating minimization of the two residuals *R*_ρ_(χ) = ∫_*V*_ [ρ(χ) − ρ(Φ)*s*_ρ_]^2^ d*V* and *R*_δ_(Φ) = ∫_*V*_*m*_ρ_[δ_*M*_(χ) − ρ(Φ)*s*_ρ_]^2^ d*V* in each iteration of the algorithm by maximizing the respective *S*_ρ_(Φ) and *S*_δ_(Φ) sum functions. While *R*_ρ_(χ) converges to zero, *R*_δ_(Φ) converges, as predicted by the theory, to a positive quantity. These two independent residuals combine δ_*M*_ and ρ each with |ρ| while keeping the same unknowns, leading to overdetermination for diffraction data extending to atomic resolution. At the beginning of a *SMAR* phase refinement, the zero part of the *m*_ρ_ mask [resulting from the zero conversion of the slightly negative ρ(Φ) values] occupies ∼50% of the unit-cell volume and increases by ∼5% when convergence is reached. The effects on the residuals of the two *SMAR* phase refinement modes, *i.e.* only using density functions (slow mode) supplemented by atomic constraints (fast mode), are discussed in detail. Due to its architecture, the *SMAR* algorithm is particularly well suited for Deep Learning. Another way of using δ-*GEQ* is by solving it in the form ρ(**r**) = δ_*M*_(**r**) − *g*(**r**), which provides a simple new derivation of the already known δ_*M*_ tangent formula, the core of the δ recycling phasing algorithm [Rius (2012). *Acta Cryst.* A**68**, 399–400]. The nomenclature used here is: (i) Φ is the set of φ structure factor phases of ρ to be refined; (ii) δ_*M*_(χ) = FT^−1^{*c*(|*E*| − 〈|*E*|〉)×exp(*i*α)} with χ = {α}, the set of phases of |ρ| and *c* = scaling constant; (iii) *m*_ρ_ = mask, being either 0 or 1; *s*_ρ_ is 1 or −1 depending on whether ρ(Φ) is positive or negative.

## Abstract 2

The general equation of the δ direct methods is established and used to define one of the two residuals of the *SMAR* phasing algorithm. These residuals utilize the absolute value of ρ and the zero conversion of slightly negative Fourier ripples (≥50% of the cell volume) and lead to overdetermination for atomic resolution diffraction data. Due to its architecture, the *SMAR* algorithm is particularly well suited for Deep Learning. Alternatively, when solved for ρ, the general equation provides a simple derivation of the already known δ_*M*_ tangent formula, the core of the δ_*M*_ recycling algorithm.

## 1. Introduction

Historically, direct methods were developed to solve small crystal structures directly from high-resolution single-crystal diffraction data. From their origins in the 1950s (Sayre, 1952 ▸; Cochran, 1952 ▸; Zachariasen, 1952 ▸), they have seen continuous advances over the years, not only driven by the steady increase in computing power, but also by clever algorithms and efficient implementations. Computing power continues to increase but the solution of larger structures at lower resolution is still hindered in the case of equal atoms and requires the use of ubiquitous model fragments and density modification. In their widespread successful application, the time scale is in any case shorter than the experimental effort involved in X-ray structure determination.

One of the most recent advances in direct methods has been the |ρ|-based algorithm (*SMAR)* maximizing the *S*_*M*,|ρ|_(= *S_MAR_*) sum function (Rius, 2020 ▸). It corresponds to the latest stage in the evolution of the *S_M_* origin-free modulus sum function (originally named *Z_R_*; Rius, 1993 ▸) which still involved triple-phase structure invariants. Even today, *Z_R_* is the simplest and certainly one of the most successful direct methods working exclusively in reciprocal space (Rius *et al.*, 1995 ▸). However, it was almost immediately superseded by the *Shake & Bake* strategy alternating between reciprocal- and real-space refinements (dual-space recycling methods) which allowed the solution of larger crystal structures (Weeks *et al.*, 1993 ▸; Miller *et al.*, 1993 ▸). Dual-space methods do not eliminate phase relationships in reciprocal space but complement them with peak picking in real space as an extreme form of density modification. A comprehensive description at the height of development of dual-space recycling methods can be found in the *International Tables for Crystallography*, Vol. F (Sheldrick *et al.*, 2012 ▸).

Following this trend, triple-phase invariants (whose number becomes exceedingly large for large structures) were replaced in *S_M_* by the more efficient and accurate Fourier transforms (FT) (Rius *et al.*, 2007 ▸; Rius, 2014 ▸). More recently, the ρ^2^ function in *S_M_* was replaced by the mathematically simpler |ρ| function and a new mask was introduced that takes the negative values of ρ into account. Both changes have led to the *SMAR* phasing algorithm (Rius, 2020 ▸). Important aspects of its application have been covered in two recent publications, the first dealing with its extension to larger crystal structures by introducing the fast inner-pixel preservation procedure (*ipp*) for density modification (Rius & Torrelles, 2021 ▸) and using initial phase values derived from the modulus function. In the second publication *SMAR* was adapted to the solution of anomalous scattering substructures in protein crystals (Rius & Torrelles, 2022 ▸). The present short introduction is intended to provide a brief overview of the 30-year development of that particular type of direct methods which shares the implicit or explicit use of the ρ ≃ δ_*M*_ (or δ_*P*_) approximation (Rius, 2012*a* ▸). To distinguish this family of direct methods from the rest – and also to help in their identification – the general term ‘δ direct methods’ is coined in this publication.

**Figure 1:**
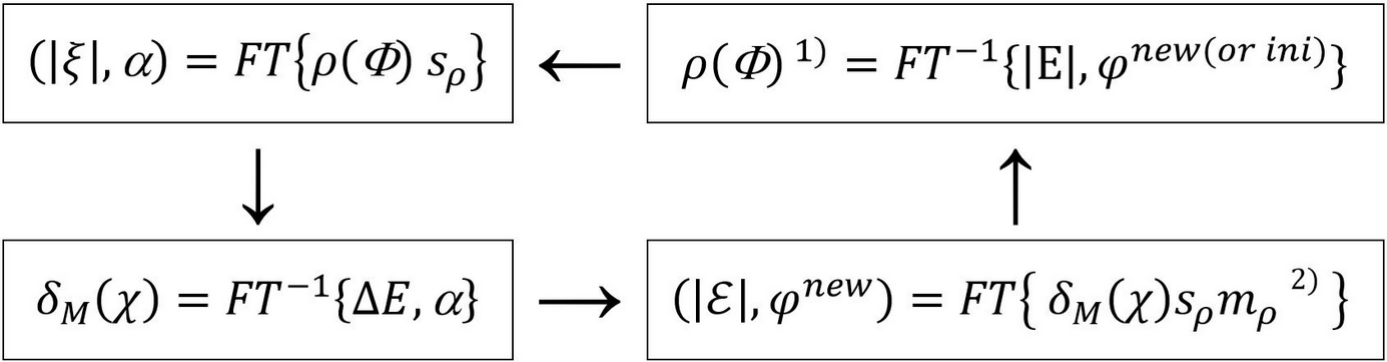
The iterative *SMAR* phasing algorithm in four stages. (Upper right-hand corner) Initial (or updated) φ phase estimates belonging to set Φ are combined with observed |*E*| values to obtain ρ and ρ(Φ)*s*_ρ_ = |ρ(Φ)| [the superscript ^1)^ indicates that ρ is stored]. (Upper left) The FT of |ρ(Φ)| is calculated to get the new set χ of α phases as well as the calculated |ξ| values. (Lower left corner) The new α values are combined with the experimental Δ*E* = |*E*| − 〈|*E*|〉 and Fourier transformed to obtain δ_*M*_(χ). The *m*_ρ_ mask and the *s*_ρ_ signs are derived from the stored ρ(Φ), and the δ_*M*_(χ)*s*_ρ_*m*_ρ_ = ρ′ product is carried out. (Lower right) The FT of ρ′ supplies the updated φ phases [the superscript ^2)^ indicates that when *ipp* is applied, ρ′ is further modified to ρ′′ before the FT operation] as well as the calculated 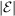 values.

The aim of this article is to complete the theoretical foundations of the *SMAR* phasing algorithm. The algorithm described by Rius (2020 ▸) and shown in Fig. 1 ▸ essentially consists of the iterative application of the phasing formula
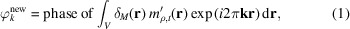
where (i) φ_*k*_ is the phase of the *k*th structure factor of ρ and belongs to the set Φ of phases to be refined; (ii) δ_*M*_(χ) is equal to 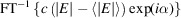 with χ = {α} being the set of phases of |ρ| and *c* a scaling constant; (iii) **r** is a position vector inside *V*, the unit-cell volume; and (iv) 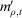 is a mask function defined in Table 1 ▸ that can take the values 1, 0 or −1. The representative test case shown in Table 1 ▸ (*t* = 2.5) indicates that the zero part in the 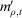 mask is around 50% at the beginning of a phase refinement with initially random phase values; as the refinement converges, the zero part increases by up to 5%. Most of the remaining part of the mask is taken up by ones, as the proportion of −1 values is kept very small (<1.0%).

The *SMAR* phasing algorithm was originally derived from the *S_MAR_* sum function. A sum function such as *S_MAR_* generally corresponds to the mixed integral of a residual as *e.g.* in the case of expression (20) in relation to (21) in this article. Only when the residual is known can the derivation of the phasing algorithm be considered complete, which leads to a better understanding of it and enables, for example, the estimation of the minimum value of the residual. In this context, the use of the δ_*M*_ ≃ ρ approximation in Rius (2020 ▸) represented a limitation. To overcome this, the relationship between δ_*M*_ and ρ is worked out in Appendix *A*[App appa], resulting in δ-*GEQ* which, when modified accordingly, leads to one of the two desired *SMAR* residuals. The derivation of the second residual is simpler and introduces the phases corresponding to |ρ(Φ)| in the algorithm. To increase the readability of this article, δ-*GEQ* is derived separately in Appendix *A*[App appa] and a summary thereof given in Section 2.2.

To complete this introduction, it is interesting to mention, particularly for newcomers, that density modification in the context of direct methods was efficiently introduced in *ACORN* (Foadi *et al.*, 2000 ▸). *ACORN* and *SHELXE* (Sheldrick, 2002 ▸) both use density sharpening and negative density elimination. Later, in the *VLD* algorithm, a difference and a flipping term were combined (Burla *et al.*, 2010 ▸). Finally, another related and more modern development was *SHELXT*, which is more broadly associated with the charge-flipping algorithm but combines it with direct methods and with density modification at part of the peak positions, used to eliminate atoms at random without atoms (Sheldrick, 2015 ▸). One distinctive feature of the density modification in *SMAR* is the zero conversion of only slight negative densities and the preservation of the inner peak pixels (Rius & Torrelles, 2021 ▸).

All calculations in this article have been performed with a modified version of the *XLENS_v1* code (Rius, 2011 ▸). The diffraction data used in the test calculations correspond to:

(i) Actinomycin Z3 with 1228 (C, N, O) + 8 Cl atoms in the unit cell. According to the refinement protocol in the Protein Data Bank (PDB code 1a7z), 4 Cl sites are partially occupied and the other 4 Cl atoms have a rather large *B* value, so that their scattering powers are considerably reduced. The minimum *d* spacing (*d*_min_) is 0.95 Å; *a* = 14.803, *b* = 24.780 and *c* = 65.059 Å, space group *P*2_1_2_1_2_1_ (Schäfer *et al.*, 1998 ▸).

(ii) Alpha1 peptide with 503 (C, N, O) + 1 Cl. *d*_min_ = 0.90 Å; *a* = 20.846, *b* = 20.909 and *c* = 27.057 Å, α = 102.40, β = 95.33 and γ = 119.62°, *P*1 (Privé *et al.*, 1999 ▸).

(iii) Pep1 with 344 (C, N, O). *d*_min_ = 1.00 Å; *a* = 13.999, *b* = 21.602 and *c* = 21.615 Å, *P*2_1_2_1_2_1_ (Antel *et al.*, 1995 ▸).

(iv) Suoa with 188 (C, N, O). *d*_min_ = 1.00 Å; *a* = 18.350, *b* = 21.441 and *c* = 8.350 Å, *P*2_1_2_1_2_1_ (Oliver & Strickland, 1984 ▸).

## 2. Basic elements of the *SMAR* algorithm

### 2.1. The ρ Fourier synthesis: its mask definition and general relationship to |ρ|

When solving crystal structures by direct methods from atomic resolution X-ray diffraction intensity data, the electron-density function is normally calculated with the Fourier synthesis
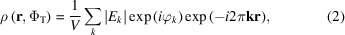
where |*E*_*k*_| is the modulus and φ_*k*_ is the phase value of the (quasi)-normalized structure factor of the *k*th reflection (Main, 1975 ▸). The moduli and phases of all reflections form the {|*E*|} and Φ = {φ} sets, respectively. For clarity, the phase type (and eventually the modulus type) used in the Fourier synthesis is added to the function name when required to avoid confusion, *e.g.* ρ(*r*, Φ_T_) specifies that ρ(*r*) is calculated with Φ_T_ (the index T stands for true phase values).

Expression (2) is stated in terms of the normalized scattering factors, *i.e.*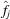 = 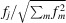 for an arbitrary atom *j* (with *f*_*j*_ being its normal scattering factor including the Debye–Waller factor). For the sake of simplicity, a crystal structure with *N* equal atoms in the unit cell is assumed throughout, so that the normalized scattering factor reduces to 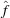 = 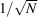.

Due to the limited number of Fourier terms (a consequence of the finite number of measured intensities), the ρ(*r*, Φ_T_) synthesis is affected by Fourier series truncation effects. These termination effects are mainly reflected in the broadening of the atomic peaks, each consisting of a large spherically symmetric positive central part (= *CORE*) surrounded by negative and positive waves (ripples) that decrease as one moves away from the peak center. For point-like atoms (for which broadening due to scattering factors and thermal vibration effects are largely removed), the limiting spherical surface of the *CORE* lies ∼0.72 × *d*_min_ (Å) from the peak center [it corresponds to the first zero of the *T*_3_ spreading function in Lipson & Cochran (1966 ▸)]. For locations lying outside neighboring *CORE*s, the ripple contributions of neighboring atoms add up and result in slightly negative and slightly positive zones (*SNZ*s and *SPZ*s, respectively). The ρ(*r*, Φ) values contained in the interval [0, −*t*σ_ρ_] make up the *SNZ*s (σ_ρ_ and *t* are defined in Table 1 ▸). Since the ρ distribution in the crystal is positive definite, the probability that the *SNZ*s accommodate the *CORE*s of atomic peaks is zero, so this information can be introduced in the form of an *m*_ρ,*t*_ mask which is 0 for all negative ρ in the [0, −*t*σ_ρ_] interval (negative ripple conversion to zero) and 1 elsewhere. As shown in Table 1 ▸, the mask value depends on ρ(*r*) and the *t* parameter (since *t* is always 2.5 in this work, it is suppressed in *m*_ρ,*t*_ for notation simplicity and we use simply *m*_ρ_). Table 1 ▸ also shows that the zero part of the mask extends to at least 50% of the unit-cell volume for *t* ≃ 2.5 (just for comparison, for *t* ≥ 10 the threshold is so low that the mask values of all negative ρ values become zero).

The relationship between the syntheses |ρ(Φ)| and ρ(Φ) is given by the equality
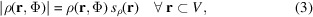
in which 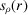 is 1 or −1 depending on whether ρ(*r*, Φ) is positive or negative. This equality is completely general, so the product ρ(**r**, Φ)*s*_ρ_(**r**) can always replace |ρ(*r*, Φ)| in the derivation of the residuals. Finally, since ρ is positive definite and diffraction data are assumed to reach atomic resolution, it is clear for Φ = Φ_T_ that the negative 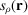 values in (3) are always associated with small ρ(*r*, Φ_T_), so that (3) becomes ρ(**r**, Φ_T_) = |ρ(**r**, Φ_T_)| 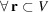. If we denote the phase set of the Fourier coefficients of the |ρ(*r*, Φ_T_)| synthesis by χ_T_, then Φ_T_ ≅ χ_T_.

### 2.2. The general equation of δ direct methods and its different forms

The δ_*M*_ Fourier synthesis defined by
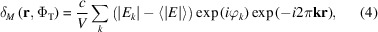
with *c* = 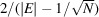, is studied in detail in Appendix *A*[App appa], showing that it contains two types of positive peak (*A* and *B*). Table 2 ▸ lists their peak strengths and positions. The stronger peaks of type *A* correspond to ρ (which are resolved in the Fourier map). The *N* − 1 times weaker peaks of type *B* are located at positions other than the atomic positions (co­incidence is accidental). These are the main constituents of the function *g*(*r*) and, due to their large number, must be severely overlapped in the unit cell. The standard form of the general equation of the δ direct methods (δ-*GEQ*) corresponds to the sum of both contributions ρ(*r*) and *g*(*r*) [equation (43)]. However, δ-*GEQ* can be used in other forms. For example it can be solved for ρ, so δ-*GEQ* then takes the form
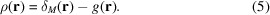


**Figure 2:**
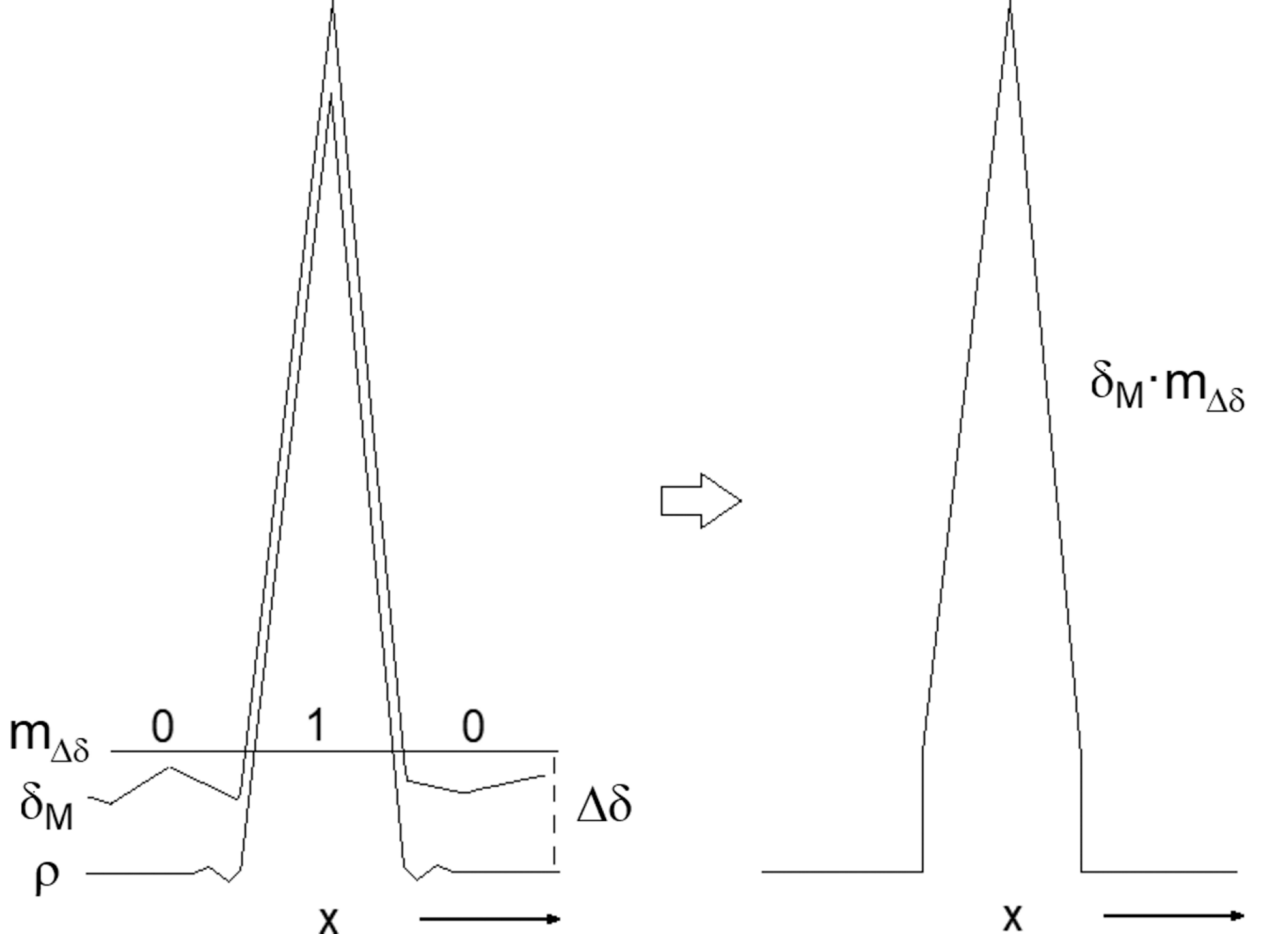
(Left) Part of a hypothetical one-dimensional unit cell corresponding to an atomic position, with schematic representation of the associated δ_*M*_(*x*) and ρ(*x*) [equation (5)]. The binary mask *m*_Δδ_(*x*) is 1 if δ_*M*_(*x*) is larger than the Δδ threshold and 0 otherwise. (Right) The same part of the unit cell shows the product function δ_*M*_(*x*)*m*_Δδ_(*x*), which is proportional to ρ(*x*) (for equiatomic structures). If the number *N* of expected atoms in the unit cell is known, then the δ_*M*_ tangent formula reduces to a structure factor calculation over the *N* largest product-function peaks greater than Δδ, *i.e.* the integral of the FT in expression (7) reduces to a sum.

One obvious difficulty here is how to handle the unknown *g* function. This difficulty can be circumvented by introducing the mask *m*_Δδ_ (being either 0 or 1), which results from the realizations that (i) δ_*M*_ and ρ have their strong peaks at the atomic positions; and (ii) *g* is formed by the positive strongly overlapped peaks of type *B* which are much more numerous but also much smaller than the peaks in δ_*M*_ and ρ. As shown in Fig. 2 ▸, the *m*_Δδ_ mask is obtained by expressing the threshold Δδ in terms of the computable σ(δ_*M*_) by means of Δδ = *t*_1_σ(δ_*M*_), with *t*_1_ ≃ 2.5, such that *m*_Δδ_(**r**) = 1 for δ_*M*_(*r*) ≥ Δδ and *m*_Δδ_(**r**) = 0 otherwise. This can be mathematically expressed by
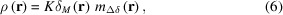
with *K* being a suitable scaling constant. Fourier transforming both sides of (6), and since *E* and ρ are linked by the Fourier transform *E* = FT(ρ), the formula
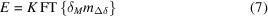
results. The angular part of (7) corresponds to the δ_*M*_ tangent formula which forms the core of the δ recycling algorithm (Rius, 2012*a* ▸,*b* ▸). It has been successfully applied to X-ray diffraction data from small crystal structures, to 3D electron diffraction data (Rius *et al.*, 2013 ▸; Capitani *et al.*, 2014 ▸) and, due to its robustness, to synchrotron tts (tts = through the substrate) microdiffraction data (Rius *et al.*, 2017 ▸). Some considerations regarding the implementation of the δ_*M*_ tangent formula are given in Section A3.

*SMAR* uses another form of δ-*GEQ* in which *g* is isolated, namely
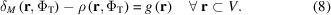
According to Φ_T_ ≅ χ_T_ at the end of Section 2.1, (8) can be expressed in terms of χ_T_ (= the set of phases corresponding to |ρ(Φ_T_)|) so that
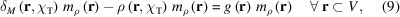
where both sides are multiplied by *m*_ρ_ [which is derived from ρ(Φ_T_)]. Expression (9) is the basic equation for one of the two *SMAR* residuals (*R*_δ_). Note the positive effect of introducing the mask *m*_ρ_. Since the zero part of the mask is ≥50% of the unit-cell volume, the unwanted contribution of *g* in (9) is suppressed for at least half of the unit cell.

## 3. The *SMAR* residuals

Each iteration of the *SMAR* algorithm consists of two differentiated parts, ending each part with the application of the corresponding phasing formula (upper-left and lower-right corners of Fig. 1 ▸). In this section, the two *SMAR* residuals leading to these phasing formulae are determined. In the following **r** is omitted unless absolutely necessary.

### 3.1. The *R*_ρ_(χ) residual

The |ρ(Φ)| density function results from applying the absolute value operator to the ρ(Φ) Fourier synthesis. The structure factors of |ρ(Φ)| correspond to its Fourier transform,
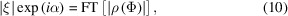
with the moduli and phase values of the structure factors being globally denoted {|ξ|} and χ, respectively. The inverse Fourier transform of both sides of (10) yields
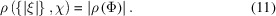
For χ = χ_T_ it can be assumed that 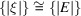. Consequently, the integral
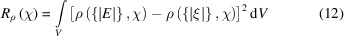
must be close to zero for 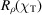 which corresponds to the minimum of *R*_ρ_. Simplifying the notation of ρ({|*E*|}, χ) to ρ(χ) and replacing ρ({|ξ|}, χ) first by |ρ(Φ)| according to (11) and then by ρ(Φ)*s*_ρ_ according to (3), integral (12) takes the simpler form
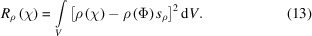


During the refinement the function ρ(Φ)*s*_ρ_ is always positive. To find the new χ set minimizing *R*_ρ_(χ), the integrand of (13) is developed into three integrals. The two integrals with integrands |ρ(Φ)|^2^ and ρ^2^(χ) are both equal to 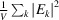 and hence phase independent; however, the third one,
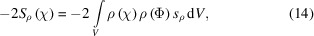
is phase dependent. The maximum of a functional like *S*_ρ_(χ) [which is equivalent to the minimum of *R*_ρ_(χ) due to the minus sign in (14)] can be found by solving the condition for an extremum, ∂*S*_ρ_/∂α = 0, ∀ α ∈ χ, which, in parallel to Rius *et al.* (2007 ▸), yields the χ phasing formula,
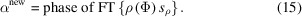


### 3.2. The *R*_δ_(Φ) residual

The residual *R*_δ_ is obtained from the left side of (9) after generalizing χ_T_ to χ. This generalization entails two changes: (i) δ_*M*_(**r**, χ_T_) is simply changed to δ_*M*_(**r**, χ), since in both cases the Fourier coefficients of δ_*M*_ contain the observed |*E*| − 〈|*E*|〉 values; and (ii) ρ(**r**, χ_T_) is changed to ρ(**r**, {|ξ|}, χ). However, since ρ(**r**, {|ξ|}, χ) = |ρ(**r**, Φ)| = ρ(**r**, Φ)*s*_ρ_(**r**) because of (11) and then (3), the selected generalized form is ρ(**r**, Φ)*s*_ρ_(**r**) [this selection ensures that ρ(Φ) enters the residual expression (17)]. By applying these two changes to the left-hand side of (9), it becomes
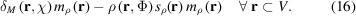
Integration of (16) after squaring gives the *R*_δ_ residual,
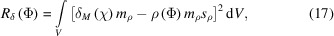
where ρ(Φ)*m*_ρ_*s*_ρ_ is always positive [= |ρ(Φ)|*m*_ρ_]. Of method­ological importance is the minimum value of *R*_δ_ which occurs for *R*_δ_(Φ_T_). An estimate of this value is obtained by squaring and integrating the right-hand side of (9), namely
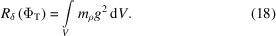
In this integral, *g* = δ_*M*_(Φ_T_) − ρ(Φ_T_) given in (8) and ρ(Φ_T_) used to calculate *m*_ρ_ are both different functions with different peak distributions. Consequently, the samples of *g*^2^ at the points where *m*_ρ_ = 1 can be assumed to be random, allowing the factorization of 〈*m*_ρ_〉 from the integral
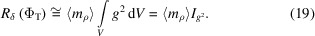
The value of the normalized 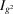 is given by equation (49) in Appendix *B*[App appb], *i.e.*
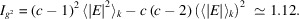
According to (19), *R*_δ_ does not converge to zero but to the positive value *R*_δ_(Φ_T_) ≃ 1.12 × 〈*m*_ρ_〉. Since it is known from Table 1 ▸ that 〈*m*_ρ_〉 is ∼0.45 (at the end of a converging refinement), the approximated value of *R*_δ_(Φ_T_) should be 0.45 × 1.12 = 0.50.

Once the residual *R*_δ_ is defined and its minimum value known, the last step is to find the Φ phase set that minimizes *R*_δ_. For this purpose, (17) is transformed into the sum
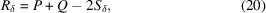
with *P*, *Q* and *S*_δ_ being the following integrals:
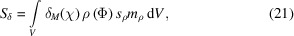

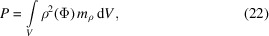

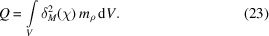


The presence of the mask *m*_ρ_ complicates the solution of these integrals. The interested reader can find in Section 3.2.1 their evaluations with the help of experimental information. The principal conclusion of Section 3.2.1 is that minimizing *R*_δ_ is essentially equivalent to maximizing *S*_δ_. Knowing this, one only needs to find the desired maximum of *S*_δ_(Φ) by solving the condition for an extremum, ∂*S*_δ_/∂φ = 0, ∀ φ ∈ Φ. By expressing ρ(Φ) in (21) as a Fourier synthesis, then
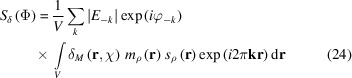
and, in parallel to Rius *et al.* (2007 ▸) and Rius (2020 ▸), the application of the condition for an extremum to (24) gives the Φ (or also *SMAR*) phasing formula,
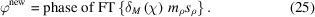
For simplicity, the Fourier-transformed function δ_*M*_*m*_ρ_*s*_ρ_ is denoted ρ′ in the slow convergence mode and ρ′′ in the fast convergence mode (see Section 4).

#### 3.2.1. Evolution of *R*_δ_, *S*_δ_, *P* and *Q* during the *SMAR* phase refinements

**Figure 3:**
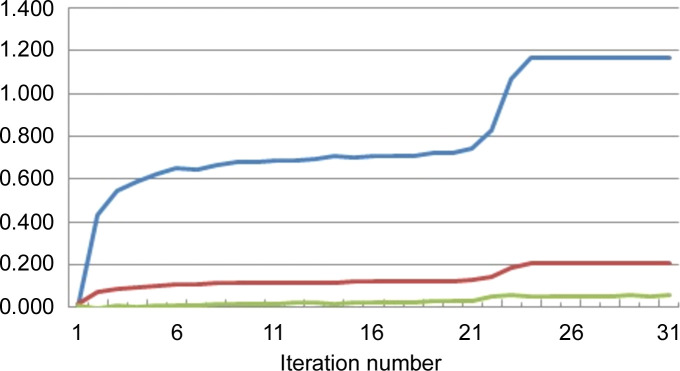
Evolution of the normalized 2*S*_*MAR*_ (top, in blue), (*P* − *P*_0_) (middle, in red) and (*Q* − *Q*_0_) (bottom, in green) for a converging *SMAR* refinement using Actinomycin Z3 data (Schäfer *et al.*, 1998 ▸) (*t* = 2.5). See the heading of Table 3 for further details.

First, the values of the integrals (21), (22) and (23) are normalized by division with
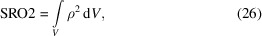
which only depends on the |*E*| values and is therefore computable. The values of −2*S*_δ_, *P* and *Q* during the phase refinement progress were determined with the *SMAR* phasing algorithm already implemented in *XLENS_v1* (Rius, 2020 ▸). The density function values used in the estimation of (21), (22) and (23) are those available before applying *ipp* (Fig. 1 ▸). Tables 3 ▸ and 4 ▸ show the evolutions for Actinomycin Z3, Suoa, Pep1 and Alpha1 peptide (each grouping of four numbers given in this subsection always refers to this order of test structures; the output files of the test calculations are available in the supporting information). For Actinomycin Z3, the evolutions of 2*S*_δ_, Δ*P* and Δ*Q* are also represented in Fig. 3 ▸. The evolution of the different integrals can be summarized as follows:

(i) Integral *S*_δ_: When starting from random phase values, the initial −2*S*_δ_ values are always close to zero for all test structures and become −1.17, −1,18, −1.16 and −1.09 at the end of the respective convergent refinements.

(ii) Integral *P*: The initial *P*_0_ value of integral *P* is 0.50 for all test structures and, as the phase refinement progresses, the difference Δ*P* = *P* − *P*_0_ increases. Δ*P* is approximately proportional to 2*S*_δ_ with the slopes 〈*k*_Δ*P*_〉 equal to 0.171 (6), 0.155 (6), 0.159 (6) and 0.162 (5) for the four test structures (Tables 3 ▸ and 4 ▸). Consequently, the following empirical linear relationship between *P* and 2*S*_δ_ can be established,
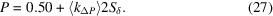


(iii) Integral *Q*: To understand the significance of integral *Q*, the integral
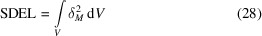
is also calculated for each test structure [it only depends on the (|*E*| − 〈|*E*|〉)^2^ quantities]. The corresponding SDEL values are 1.762, 1.790, 1.756 and 1.728. According to Tables 3 ▸ and 4 ▸, the initial *Q* values are *Q*_0_ = 0.89, 0.89, 0.88 and 0.89, *i.e.Q*_0_ ≃ 0.50 × SDEL. In addition, during the respective phase refinements, the largest *Q* − *Q*_0_ differences are only 0.05, 0.01, 0.02 and 0.01. Consequently, it can be assumed that 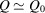.

By taking all these results into account, *R*_δ_ can be simplified to
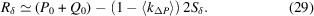
Since *P*_0_, *Q*_0_ and 〈*k*_Δ*P*_〉 can be considered nearly constant during the phase refinement, it follows from (29) that minimizing *R*_δ_ is essentially equivalent to maximizing *S*_δ_.

## 4. The phase refinement modes in *SMAR*

Since most experimental results of *SMAR* have already been discussed by Rius & Torrelles (2021 ▸, 2022 ▸) and in Section 3.2.1 of this contribution, only a selection of points directly related to the topic of this article are treated here, grouped according to the convergence mode.

### 4.1. The slow convergence mode

This mode only works with density functions, that is, the positions of the atomic peaks are not used. This mode requires the inclusion of the Fourier terms of all reflections (strong + weak) in the calculation of ρ and δ_*M*_. The ρ′ = δ_*M*_*m*_ρ_*s*_ρ_ values entered in the *SMAR* phasing formula are obtained as follows:

(i) For slightly negative ρ values (amounting to 50–55% of the unit cell), the ρ′ values are 0.

(ii) For positive ρ values (regardless of their strength and representing 45–50% of the unit cell), ρ′ is equal to δ_*M*_.

(iii) Only for very negative ρ values (<1% of the unit cell for *t* ≃ 2.5), ρ′ is equated to −δ_*M*_ (the minus sign multiplying δ_*M*_ tends to restore the sign of the very negative ρ value).

In summary, ρ′ corresponds either to unrestricted δ_*M*_ and −δ_*M*_ values or to fixed δ_*M*_ = 0 ones. The mask used by the *SMAR* algorithm results in smooth phase refinements based only on density functions. On the other hand, the experimental *R*_δ_(Φ_T_) values calculated at the end of converging phase refinements using (20) are 0.48, 0.42, 0.43 and 0.47 for Actinomycin Z3, Suoa, Pep1 and Alpha1 peptide, respectively (Tables 3 ▸ and 4 ▸). These values agree with the theoretical estimation of *R*_δ_(Φ_T_) ≃ 0.50 found in Section 3.2.

Regarding the very negative densities, some of them are due to the wrong input model (generated by the random starting phases) and disappear during the convergence process. The reductions observed in ρ′ are 0.17% → 0.08% for Actinomycin Z3, 0.18% → 0.06% for Alpha1 peptide, 0.18% → 0.02% for Pep1 and 0.17% → 0.01% for Sucrose (Tables 3 ▸ and 4 ▸). To get an idea of the effect of *t* on the phasing of the intensity data of the four test structures, the sums of their total number of successful trials (out of 25) were determined for *t* = 1.5, 2.0, 2.5 and 10.0 (in the last case, the negative densities are all zero). The respective sums are 34, 76, 69 and 59 (Table S1 in the supporting information). The largest sums are obtained for *t* = 2.0 and 2.5 and the smallest for *t* = 1.5. For *t* = 10.0, the resulting sum is slightly worse than for *t* = 2.0 or 2.5. These results suggest that (i) the best *t* values are between 2.0 or 2.5, and (ii) although they represent only a small percentage of the unit cell, setting the very negative densities equal to zero is not beneficial for phase refinement. A possible explanation of the physical meaning of the very negative densities can be found in Section 3.3 of Rius (2020 ▸). In any case, a future comprehensive study focusing on this point would be useful.

### 4.2. The fast convergence mode

In this mode, which is the default operating mode of the *SMAR* algorithm, the part working only with density functions is supplemented by an additional step in which ρ′ is modified by the *ipp* method to give ρ′′ (Rius & Torrelles, 2021 ▸), which in turn replaces ρ′ in the Φ phasing formula (25). The *ipp* method is an effective way of accelerating the phase refinement and assumes that the approximate number *N* of expected atoms is known (which is normally the case). Briefly explained, *ipp* identifies in the ρ′ Fourier map those grid points closest to the centers of the *N* largest peaks. The ρ′ values of the 27 inner-peak grid points are then preserved for each peak and the remaining grid points of the ρ′ Fourier map are set to zero, giving rise to the new ρ′′. In this way no interpolation is required to find peak centers and, at the same time, the large ρ′ values are preserved. Additionally, if the grid size Δ_grid_ is ∼0.33 Å, *ipp* implicitly applies the minimum interpeak separation (*mips*) constraint. The criterion for considering a positive maximum of the ρ′ Fourier synthesis a peak is that the voxel closest to the peak center must be surrounded by 26 smaller nearest-neighbor voxels (some can even be negative). Consequently, none of the nearest neighboring voxels can become the center of another ρ′ peak. This means that for an isometric grid element with Δ_grid_ = 0.33 Å the average *mips* value is 0.955 Å [the minimum, intermediate and maximum separations are 2 × 0.33 × 1 = 0.67 Å (6×), 2 × 0.33 × 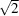 = 0.96 Å (12×) and 2 × 0.33 × 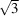 = 1.16 Å (8×), respectively]. According to (19), the value of *R*_δ_(Φ_T_) is proportional to 〈*m*_ρ_〉. In the fast convergence mode, due to the application of *ipp*, 〈*m*_ρ_〉 = 27*N*/*N*_vox_, where *N*_vox_ is the total number of voxels in the unit cell. For Actinomycin Z3, 27*N* = 33372 and *N*_vox_ = 664875, and hence 〈*m*_ρ_〉 = 0.05, much smaller than the typical 〈*m*_ρ_〉 values for the slow convergence mode (∼0.45). Accordingly, *R*_δ_(Φ_T_) = 1.12 × 0.05 = 0.06 is also much smaller than in the slow convergence mode.

A characteristic of this mode is that the calculation of ρ(Φ) only includes Fourier terms of those reflections satisfying the |*E*| ≥ |*E*|_lim_ condition with |*E*|_lim_ = 1.0, while the calculation of δ_*M*_(χ) (and thus of ρ′) is always done with the Fourier terms of all *k* reflections (Fig. 1 ▸). That only the large |*E*| values should participate in the ρ update is certainly related to the fact that only the 27 inner voxels close to the peak center are preserved (the rest of the peak voxels become part of the zero mask). This is supported by the fact that, in the slow convergence mode, the Fourier terms of all reflections must be included in the calculation of ρ(Φ).

### 4.3. The correlation coefficient

In addition to estimating *R_δ_*, the agreement of minuends and subtrahends in (17) can also be estimated using the correlation coefficient
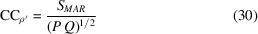
(using the density values before *ipp* for the slow convergence case). For refinements reaching convergence, the found CC_ρ′_ values are 0.715 for Actinomycin Z3 (Table 3 ▸), 0.742 for Suoa, 0.736 for Pep1 and 0.705 for Alpha1 peptide (Table 4 ▸). These moderately high correlation coefficients also confirm the small discrepancy introduced in the CC_ρ′_ calculation by the *g* contributions of those voxels with *m*_ρ_ = 1. As expected, the corresponding CC_ρ′′_ values (also listed in Tables 3 ▸ and 4 ▸) are much higher due to the smaller number of voxels with *m*_ρ_ = 1, *e.g.* CC_ρ′_ = 0.72 and CC_ρ′′_ = 0.90 for Actinomycin Z3.

## 5. Conclusions

The main objective of this research was to complete the theoretical aspects of the *SMAR* phasing algorithm. For this purpose, the connection between δ_*M*_ and ρ has been examined in detail. This leads to the general equation of δ direct methods (δ-*GEQ*) which, written in its standard form, is δ_*M*_ = ρ + *g*, where the density function *g* is mainly formed by a large number of small positive *B*-type peaks. Two ways of using δ-*GEQ* have been investigated. In *SMAR*, δ-*GEQ* is used in its difference form, δ_*M*_ − ρ = *g*, while in δ recycling it is used solved for ρ, so ρ = δ_*M*_ − *g*. In this second case, it has been shown that the δ_*M*_ tangent formula can be derived directly from it by including a suitable mask.

Regarding the *SMAR* residuals it can be concluded that:

(i) *R*_ρ_(χ) measures the [ρ(χ) − ρ(Φ)*s*_ρ_]^2^ differences in the entire unit cell. The minimum value of *R*_ρ_ is *R*_ρ_(χ_T_) ≃ 0.

(ii) The *R*_δ_(Φ) residual is based on its basic equation (9), *i.e.* δ_*M*_(χ_T_)*m*_ρ_ − ρ(χ_T_)*m*_ρ_ = *gm*_ρ_, where χ_T_ are the α phases corresponding to |ρ(Φ_T_)|.

(iii) *R*_δ_(Φ) measures the [δ_*M*_(χ)*m*_ρ_ − ρ(Φ)*m*_ρ_*s*_ρ_]^2^ differences in the entire unit cell after considering that ρ(χ) ≅ ρ(Φ)*s*_ρ_. The minimum value corresponds to *R*_δ_(Φ_T_) 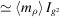 where 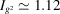. It is shown that minimizing *R*_δ_(Φ) is essentially equivalent to maximizing the sum function *S*_δ_(Φ) [equation (21)], despite the presence of the *m*_ρ_ mask in the *R*_δ_(Φ) definition. In all the examples calculated by the author, the *S*_δ_ maximum (characterized by a sudden *S*_δ_ increase) is always the true solution Φ_T_ which stands out clearly from the false solutions.

(iv) The convergence of *SMAR* is achieved by alternately applying the χ and Φ (or *SMAR*) phasing formulas in each iteration. These are α^new^ = phase of FT{ρ(Φ)*s*_ρ_} and φ^new^ = phase of FT{δ_*M*_(χ)*m*_ρ_*s*_ρ_}, respectively.

It has been found that at the start of a *SMAR* refinement, the zero part of the mask (created by converting the slightly negative density function values to zero) occupies 50% of *V* and increases by ∼5% after convergence. According to *R*_δ_(Φ_T_) ≃ 1.12〈*m*_ρ_〉 the presence of the zero part of the mask leads to a drop in the *R*_δ_ value when convergence begins, since the volume of the regions with only a *g* contribution is reduced. When the number *N* of expected atoms is actively used (fast convergence mode), each *SMAR* iteration is supplemented with the *ipp* application, which increases significantly the volume of the zero part of the mask.

Finally, a brief reflection is in order. It is known that non-crystalline materials have a continuous diffraction pattern and that oversampling of the intensity data (Shannon, 1949 ▸) results in an overdetermined system of equations from which the phases can be solved (even at non-atomic resolution) (Miao *et al.*, 2000 ▸). It is also known that oversampling cannot be applied to crystals due to their 3D periodicity. Here it is shown that, in the case of crystals, the combination of δ_*M*_ and ρ each with |ρ| produces two independent residuals while keeping the same unknowns. This also leads to overdetermination which should explain the observed efficiency of *SMAR*. It is also interesting to note that the *SMAR* algorithm is particularly well suited for Deep Learning due to its architecture.

## Supplementary Material

Table S1 and output of test calculations of Section 3.2.1. DOI: 10.1107/S2053273324009628/tw5010sup1.pdf

## Figures and Tables

**Table 1:** Values of the *m*_ρ_(**r**) mask and the *s*_ρ_(**r**) sign functions obtained from ρ(**r**, Φ) and for *t* = 2.5 with 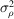 being the (phase-independent) variance of ρ(**r**) and 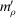 = *s*_ρ_*m*_ρ_ (Rius, 2020 ▸) The meanings of *CORE*s, *SPZ*s and *SNZ*s are explained in Section 2.1. Columns 6 and 7 give the mask compositions in % at the first and last iterations of a *SMAR* phase refinement reaching convergence (slow convergence mode) using the diffraction data for Actinomycin Z3 (Schäfer *et al.*, 1998 ▸).

Condition	Corresponds to	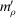	*m*_ρ_	*s*_ρ_	First	Last
ρ(**r**, Φ) > 0	*CORE*s and *SPZ*s	1	1	1	50.0	45.3
0 ≤ ρ(**r**, Φ) > −*t*σ_ρ_	*SNZ*s	0	0	−1	49.4	54.5
ρ(**r**, Φ) ≤ −*t*σ_ρ_	Very negative values	−1	1	−1	0.62	0.23

**Table 2:** Overview of the properties of the two main peak types of the δ_*M*_ Fourier synthesis The peak strength of a ρ(**r**_*l*_) peak corresponds to *fN*_ref_/*V*.

Peak type (function)	Peak positions	Peak strengths	Number in unit cell
*A* → ρ	At **r**_*l*_ atomic positions (*l* = 1, *N*)	ρ(**r**_*l*_) = δ_*M*_(**r**_*l*_)	*N*
*B* → *g*	At **r**_*jlm*_ = **r**_*j*_ + **r**_*l*_ − **r**_*m*_ with *l* ≠ *m* and *j* ≠ *m* (*j*, *l*, *m* = 1, *N*)	*g*(**r**_*jlm*_) ≃ strength of {ρ(**r**_*l*_)/(*N* − 1)}	*N*(*N* − 1)^2^

**Table 3:** Evolution of −2*S*_δ_ [equation (21)], *P* [equation (22)], *Q* [equation (23)] and *R*_δ_ [equation (20)] during a converging default *SMAR* refinement from random starting phases for Actinomycin Z3 (Schäfer *et al.*, 1998 ▸), normalized by division by SRO2 [equation (26)] (*t* = 2.5) 〈*k*_Δ*P*_〉 in (27) is the proportionality constant *k*_Δ*P*_ averaged over the number of refinement cycles. The columns headed 0 and −1 list the number of zero and negative voxels, respectively, in the ρ′ map in %. The columns headed CC_ρ′_ and CC_ρ′′_ give the correlation coefficients before and after the *ipp* application.

Iteration	−2*S*_δ_	*P*	*Q*	*R*_δ_	*k*_Δ*P*_	0	−1	CC_ρ′_	CC_ρ′′_
1	0.000	0.518	0.888	1.41	–	49.75	0.17	0.002	0.515
2	−0.436	0.574	0.880	1.02	0.169	51.60	0.09	0.307	0.652
5	−0.622	0.601	0.890	0.87	0.162	52.32	0.08	0.425	0.710
10	−0.678	0.614	0.898	0.83	0.168	52.39	0.10	0.457	0.729
15	−0.700	0.621	0.905	0.82	0.172	52.48	0.10	0.467	0.745
20	−0.724	0.624	0.913	0.81	0.172	52.53	0.11	0.479	0.746
21	−0.744	0.628	0.915	0.80	0.172	52.62	0.10	0.491	0.767
22	−0.826	0.642	0.936	0.75	0.172	52.91	0.09	0.533	0.800
23	−1.070	0.687	0.944	0.56	0.175	55.00	0.07	0.664	0.876
24	−1.164	0.710	0.936	0.48	0.172	54.65	0.08	0.714	0.906
25	−1.169	0.710	0.934	0.48	0.172	54.70	0.07	0.717	0.908
29	−1.166	0.710	0.938	0.48†	0.180	54.68	0.08	0.715	0.903
					〈*k*_Δ*P*_〉 = 0.171 (6) (28×)				

† *R*_δ_ value at the end of the converging refinement.

**Table 4:** As for Table 3 ▸ but for three additional test examples Only three stages of each phase refinement have been selected (at the beginning, when convergence begins and when it ends). In all three examples *Q* − *Q*_0_ ≤ 0.02 during the phase refinement.

Data set	Iteration	−2*S*_δ_	*P*	*Q*	*R*_δ_	*k*_Δ*P*_	0	−1	CC_ρ′_	CC_ρ′′_
Suoa	1	−0.033	0.517	0.893	1.38	–	49.71	0.17	0.024	0.602
(Oliver & Strickland, 1984 ▸)	13	−0.747	0.617	0.890	0.76	0.157	53.62	0.04	0.504	0.753
	27	−1.180	0.702	0.900	0.42	0.171	56.44	0.01	0.742	0.917
						〈*k*_Δ*P*_〉 = 0.155 (6) (26×)				
										
Pep1	1	−0.025	0.520	0.880	1.38	–	49.86	0.18	0.018	0.602
(Antel *et al.*, 1995 ▸)	14	−0.712	0.611	0.875	0.77	0.154	53.13	0.05	0.487	0.740
	28	−1.169	0.702	0.899	0.43	0.173	55.76	0.02	0.736	0.907
						〈*k*_Δ*P*_〉 = 0.159 (6) (27×)				
										
Alpha1 peptide	1	−0.013	0.519	0.866	1.37	–	49.90	0.18	0.000	0.575
(Privé *et al.*, 1999 ▸)	21	−0.651	0.605	0.861	0.82	0.156	52.40	0.09	0.451	0.711
	42	−1.089	0.687	0.875	0.47	0.167	54.18	0.05	0.703	0.906
						〈*k*_Δ*P*_〉 = 0.162 (5) (41×)				

## Appendix A Derivation of the general equation of δ direct methods

### A1. Definition and peak analysis of the δ_*M*_ Fourier synthesis

The term δ_*M*_ is defined in the unit cell by

with {φ} = Φ_T_ and with *c* being an appropriate scaling constant. It can be reformulated by making use of the δ_*M*_ = δ_*P*_/2 equality (Rius, 2012*a* ▸), wherein δ_*P*_ is the Fourier synthesis which differs from (31) only in that (|*E*_*k*_| − 〈|*E*|〉) is replaced by (|*E*_*k*_|^2^ − 〈|*E*|^2^〉). Consequently, (31) can be written as

By expressing (|*E*_*k*_|^2^ − |*E*|^2^) and  in terms of atom positions, it follows that

with  denoting the normalized scattering factor of atom *j*. As can be derived from (33), δ_*M*_ has *N*^3^ − *N*^2^ peaks at **r** = **r**_*j*_ + **r**_*l*_ − **r**_*m*_ (*l* ≠ *m*), since  is the unit for all *k*. Of interest are the values of δ_*M*_ at the *N* atomic positions, *i.e.* at **r** = **r**_*l*_ with *l* = 1, *N*. It can easily be verified that there are *N* − 1 superposed peaks contributing to δ_*M*_(**r**_*l*_), *i.e.* those that satisfy the equation **r**_*l*_ = **r**_*j*_ + **r**_*l*_ − **r**_*m*_ with *l* ≠ *m* and *j* = *m*, *e.g.* for *N* = 3 and **r**_2_ these are **r**_1_ + **r**_2_ − **r**_1_ and **r**_3_ + **r**_2_ − **r**_3_. To estimate the total strength of the δ_*M*_ peak at **r** = **r**_*l*_, expression (33) is rearranged into

with

For *r* = *r*_*l*_, the  term in (34) becomes unity. In addition, if (35) and (36) are considered, expression (34) can be further simplified to

since  = 〈|*E*|〉_*k*_*N*_*k*_ and, for an equal atom structure, it holds that

The strength of an atomic peak in ρ is , so (38) can be simplified to

by considering  = . Finally, by making

the strength of δ_*M*_(**r**_*l*_) is equal to ρ(**r**_*l*_) in (40). The δ_*M*_ peaks placed at atomic positions compose the set of type *A* peaks.

Let us consider the remaining δ_*M*_ peaks at **r** = **r**_*j*_ + **r**_*l*_ − **r**_*m*_ with *l* ≠ *m* and *j* ≠ *m*, which form the set of type *B* peaks. For a given *B* peak, the corresponding **r** position is obtained by adding the **r**_*j*_ − **r**_*m*_, *j* ≠ *m*, interatomic vector to the atomic position vector **r**_*l*_, so the superposition of **r** with an atomic position is accidental. Consequently, the strength of a (single) *B*-type peak, *e.g.* at **r**_*jlm*_, is weaker than that of a δ_*M*_(**r**_*l*_) peak (formed by the superposition of *N* − 1 single peaks). According to (40), it holds that

### A2. The general equation for δ_(*M*)_ direct methods

According to the above results, δ_*M*_(Φ_T_) contains two types of positive peaks (*A* and *B*). Table 2 ▸ lists their peak strengths and positions. The stronger peaks of type *A* correspond to ρ and are resolved in the Fourier map. The *N* − 1 times weaker peaks of type *B* are located at positions other than the atomic positions (co­incidence is accidental). Due to their large number, *i.e.**N*(*N* − 1)^2^ [for comparison, there are only *N*(*N* − 1) non-origin peaks in the modulus function], these peaks must have a strong overlap in the unit cell. The peaks of type *B* are the main constituents of the *g* function. Consequently, δ_*M*_ can be considered as the sum of both contributions, *i.e.*

which is called the general equation of δ_(*M*)_ direct methods. In the following, the subscript *M* is left out of the equation name for the sake of generality. (Note that the *g* function could also contain contributions from even weaker unconsidered peaks.)

### A3. The δ_*M*_ Fourier synthesis expressed with the signs of the |*E*| − 〈|*E*|〉 differences in the phase terms

In general, the Fourier coefficients of δ_*M*_ and ρ have different moduli and may differ in phase values. This is not obvious when comparing (2) and (4) because in both expressions the corresponding Fourier terms have the same φ phase values (which is quite useful for programming). However, when the absolute values ||*E*| − 〈|*E*|〉| are used in the synthesis, a phase shift Δφ must be added to each φ to take into account the sign. Consequently, while the Fourier coefficients of ρ are |*E*|exp(*i*φ), those of δ_*M*_ are ||*E*| − 〈|*E*|〉|exp[*i*(φ + Δφ)], with Δφ = 0 for |*E*| > 〈|*E*|〉 (strong reflections) and Δφ = π for |*E*| < 〈|*E*|〉 (weak reflections).

## Appendix B Solution of integral *I*_*g*^2^_ =∫_*V*_ *g*^2^ d*V*

Assuming that the integrand of  is *g*^2^ = [δ_*M*_(Φ_T_) − ρ(Φ_T_)]^2^, squaring the binomial gives

The integral of the first two summands in (44) when expressed in terms of the respective Fourier coefficients is

If one proceeds analogously with the third summand in (44), it follows that

This means that the integral is zero except for *k*′ = −*k* where it becomes *V*, so that the final term in (46) can be approximated by

The values of the exponentials in (47) are 1, so that it reduces to

By adding (45) and (48) and normalizing by  =  = , the final expression of the integral  is obtained, namely

From the theory of the distribution of |*E*| values, the values of 〈|*E*|^2^〉, 〈|*E*|〉 (acentric case) and *c* ≃ 2/〈|*E*|〉 can be derived, *i.e.* 1.00, 0.89 and 2.25, respectively, so that (49) gives  ≃ 1.12. Note that  only depends on |*E*| and |*E*| − 〈|*E*|〉, since the phases cancel out. Consequently, the values of  for *g*^2^ equal to [δ_*M*_(Φ_T_) − ρ(Φ_T_)]^2^ and to [δ_*M*_(χ_T_) − ρ(χ_T_)]^2^ are identical.
