# Supplementary material for: The general equation of δ direct methods and the novel SMAR algorithm residuals using the absolute value of ρ and the zero conversion of negative ripples
Source: Acta Crystallogr A Found Adv. 2025 Jan 1;81(Pt 1):16–25. doi: 10.1107/S2053273324009628 (PMC11694219; doi:10.1107/S2053273324009628)
Supplement: Supplementary file 1 [file a-81-00016-sup1.pdf]

FOUNDATIONS
ADVANCES

Volume 81 (2025)

Supporting information for article:

The general equation of δ direct methods and the novel *SMAR* algorithm residuals using the absolute value of ρ and the zero conversion of negative ripples

Jordi Ruis

TABLE S1. Number of successful trials (out of 25) obtained with the mask scheme in Table 1 for t = 1.5, 2.0, 2.5 and 10.0 (all negative densities are set to zero). All *SMAR* phase refinements were performed in *P1* starting with random phase values. N_{atom} = number of non-H atoms in the unit cell. *Max* n_{cycles} is the maximum number of permitted cycles per trial. Bottom row: sum of the number of successful trials of the 4 test structures.

Code	N_{atom}	t=1.5	t =2.0	t = 2.5	t = 10.0	*Max* n_{cycles}
Suoa[(a)]	188	12	16	11	8	100
Pep1[(b)]	340	2	20	18	17	150
Alpha1-peptide[(c)]	479 + Cl	20	21	18	18	400
Actinomycin Z3[(d)]	1228 + 8Cl	0	19	22	16	400
∑		34	76	69	59	

References: (*a*) Oliver & Strickland (1984); (*b*) Antel et al. (1995); (*c*) Privé et al. (1999); (*d*) Schäfer et al. (1998)

OUTPUT OF TEST CALCULATIONS

```
============================================================
   XLENS_SMARV241: A DELTA DIRECT-METHODS PROGRAM
   Crystal structure solution with the SMAR phasing algorithm
   Copyright Prof. Jordi Rius (Palleiro)
   Rius J. Acta Cryst (2020) A76 489-493
   Institut de Ciencia de Materials de Barcelona (CSIC)
   Date is 19- 2-2024   at 19:37:51
 ============================================================


     ACTINOMICIN  P212121    8 pdb_sf 1A7Z

    Crystal data:
     a =   14.803 =     alpha=   90.000
     b =   24.780 =     beta=    90.000
     c =   65.059 =     gamma=   90.000

     Reciprocal lattice constants (a, b, c):
     (sinth/l)2=  0.001141*H2 + 0.000407*K2 + 0.000059*L2 +
                 0.000000*HK + 0.000000*HL + 0.000000*KL

     Unit cell volume (a,b,c) =     23864.84

     Unit cell (a,b,c) is P centered
      nodes at:
       1)  0.000000       0.000000       0.000000

      Symmetry operations:
      R11  R12  R13     R21  R22  R23     R31  R32  R33    T1     T2     T3
  1)    1    0    0       0    1    0       0    0    1    0.00   0.00   0.00
  2)   -1    0    0       0   -1    0       0    0    1    0.50   0.00   0.50
  3)   -1    0    0       0    1    0       0    0   -1    0.00   0.50   0.50
  4)    1    0    0       0   -1    0       0    0   -1    0.50   0.50   0.00

          Unit cell contents
      Symbol  Atomic_number    Number in cell    type    scat_power
        CL         17               8            1         17.00
        C           6            1224            2          6.00
        H           1             400            3          1.00

     X-RAYS
```

```
     RANDOM starting phases

--------------------------------------------------------------

     Intensity data information:
     ==========================

     Input data are F**2
     DSMIN (Angs.):            0.9520
     DSFOU (Angs.):            0.9520
--------------------------------------------------------------

     FORBIDDEN REFLECTIONS IN DATA FILE *.HKL:
     =========================================

    H    K    L        XO       DXO      NEQ
--------------------------------------------------------------

     Rsigma(I) of equivalent reflections
     Rsig:    0.00000  Nref:         0

     ds_interval        Rsigma       Nref

     N. UNIQUE REFLECTIONS (measured,theory):       15708       15800


     INFORMATION OF DATA IN P1:
     MEAS. INTENSITIES (OBS+UNOBS)IN XOHEM.HKL=        57723
     NOT MEASURED INTENSITIES in XOUNMEAS.HKL=         262
     FRACTION MEASURED INTENSITIES >DSFOU:     0.995
     HMAX=   15  KMAX=   26  LMAX=   68


--------------------------------------------------------------

     SCALING OF MEASURED INTENSITIES:
     ================================


      SCALE FACTOR:        0.9920  F(EXP)/SK = F(ABS)
      B OVERALL   :        4.3351
      R(Wilson)   :       49.7681
      DSFOU       :        0.9520
      FACDF       :        2.0000  Observed if XO > FACDF*sig(XO)
```

SHELL	DMEAN	DINF	SFC2/SFO2	Nref	f.unobs
1	2.05	1.63	0.85862	11557	0.0675
2	1.42	1.29	1.44632	11637	0.0479
3	1.20	1.13	0.86641	11483	0.0543
4	1.07	1.03	0.83442	11688	0.1757
5	0.99	0.95	1.11385	11358	0.4018

DATA NORMALIZING:
================

NUMBER OF INPUT REFLEXIONS = 57723
F(ABS) = F(EXP) / 0.9920

	CENT	ACENT	HK0	0KL	H0L	REST
</E2-1/>	0.968	0.736	1.036	0.900	0.968	0.748

E2MEAN : 0.963639

<E**2> ACCORDING TO PARITY GROUPS (D-SPACING > 0.952

	ALL	GGG	GGU	GUG	GUU	UGG	UGU	UUG	UUU
E^2	1.000	0.953	0.984	1.008	0.982	1.037	1.030	1.014	0.992
N	57723	7188	7206	7209	7220	7216	7228	7240	7216

PHASE REFINEMENT DETAILS:
========================

TOTAL N. OF E-VALUES = 57723
D-SPACING CUT-OFF = 0.95
<E>, <E2> = 0.8701 1.0000

N. SETS AND CYCLES = 25 29
ELIM FOR K REFLECTIONS = 0.00
N.REFLECTIONS > EKLIM = 57723
ELIM FOR H REFLECTIONS = 1.00
N.REFLECTIONS > EHLIM = 19502
N. ATOMS IN UNIT_CELL (NCELL) = 1232
PIXELS(0); P_ATOMS(1); OMIT(2) = 1
FRAC. RANDOMLY DELETED ATOMS = 0.000

GRID SPACING IN ANGS. = 0.33
N.GRID POINTS (XYZ) = 40 72 196

LOWER SDV FACTOR FOR MASK = -2.50
ESD FACT FOR PEAK ACCEPTANCE (ATOMS=1,2) = 3.70
ESD FACTOR IN SEARCH (DIF.FOURIER) = 1.10

ITER	SRO2	SDEL2	2S_RDM	P	Q	RDEL	%0	%-1	CCro’	CCro”
1	0.475E+11	0.837E+11	-0.0002	0.5176	0.8876	1.4049	49.746	0.174	0.0002	0.5154
2	0.475E+11	0.837E+11	-0.4357	0.5738	0.8800	1.0182	51.595	0.092	0.3065	0.6518
3	0.475E+11	0.837E+11	-0.5448	0.5853	0.8873	0.9278	52.147	0.068	0.3780	0.6850
4	0.475E+11	0.837E+11	-0.5865	0.5938	0.8870	0.8944	52.295	0.075	0.4041	0.6978
5	0.475E+11	0.837E+11	-0.6215	0.6008	0.8897	0.8690	52.323	0.079	0.4250	0.7103
6	0.475E+11	0.837E+11	-0.6535	0.6069	0.8926	0.8460	52.447	0.086	0.4439	0.7206
7	0.475E+11	0.837E+11	-0.6467	0.6074	0.8927	0.8534	52.354	0.092	0.4391	0.7227
8	0.475E+11	0.837E+11	-0.6689	0.6125	0.8972	0.8408	52.414	0.095	0.4512	0.7198
9	0.475E+11	0.837E+11	-0.6777	0.6127	0.8959	0.8310	52.453	0.091	0.4573	0.7233
10	0.475E+11	0.837E+11	-0.6784	0.6139	0.8981	0.8336	52.391	0.097	0.4568	0.7288
11	0.475E+11	0.837E+11	-0.6847	0.6149	0.8982	0.8285	52.409	0.092	0.4606	0.7346
12	0.475E+11	0.837E+11	-0.6875	0.6177	0.9068	0.8371	52.414	0.095	0.4593	0.7272
13	0.475E+11	0.837E+11	-0.6912	0.6175	0.9014	0.8277	52.462	0.093	0.4632	0.7393
14	0.475E+11	0.837E+11	-0.7071	0.6189	0.9012	0.8129	52.520	0.090	0.4734	0.7438
15	0.475E+11	0.837E+11	-0.7002	0.6205	0.9045	0.8248	52.478	0.103	0.4673	0.7449
16	0.475E+11	0.837E+11	-0.7111	0.6223	0.9045	0.8158	52.470	0.105	0.4739	0.7409
17	0.475E+11	0.837E+11	-0.7051	0.6231	0.9074	0.8254	52.431	0.114	0.4689	0.7410
18	0.475E+11	0.837E+11	-0.7072	0.6216	0.9046	0.8191	52.409	0.099	0.4715	0.7397
19	0.475E+11	0.837E+11	-0.7197	0.6239	0.9087	0.8128	52.496	0.106	0.4779	0.7456
20	0.475E+11	0.837E+11	-0.7238	0.6244	0.9125	0.8131	52.525	0.106	0.4795	0.7461
21	0.475E+11	0.837E+11	-0.7435	0.6276	0.9146	0.7988	52.619	0.095	0.4906	0.7674
22	0.475E+11	0.837E+11	-0.8260	0.6422	0.9360	0.7523	52.908	0.094	0.5327	0.8001
23	0.475E+11	0.837E+11	-1.0697	0.6867	0.9435	0.5604	53.996	0.068	0.6645	0.8759
24	0.475E+11	0.837E+11	-1.1638	0.7098	0.9360	0.4819	54.649	0.076	0.7139	0.9064
25	0.475E+11	0.837E+11	-1.1688	0.7102	0.9341	0.4756	54.701	0.074	0.7175	0.9082
26	0.475E+11	0.837E+11	-1.1690	0.7105	0.9350	0.4765	54.740	0.075	0.7171	0.9053
27	0.475E+11	0.837E+11	-1.1677	0.7101	0.9359	0.4783	54.641	0.075	0.7162	0.9031
28	0.475E+11	0.837E+11	-1.1662	0.7095	0.9358	0.4791	54.634	0.072	0.7156	0.9018
29	0.475E+11	0.837E+11	-1.1656	0.7102	0.9378	0.4825	54.642	0.078	0.7141	0.9029

SUMMARY OF SOLUTIONS:

SET	CCH	SEED	CCK	NCYC	NITER	RVAL(ini,end)		PKS(ini,end)		RO/SIG
1	0.9164	0.5769230	0.8336	29	3	29.2	18.8	469	345	22.28

TOTAL NUMBER OF CYCLES IS 29 IEXCC = 0

PEAK SEARCH:
===========

SOLUTION N.   1 R =   18.777130 ITERATIONS=   3

NUMBER OF E AND CUT-OFF VALUE   8959   0.70
GRID: NX= 40 NY= 72 NZ= 196 SIZE(ANGS)= 0.33
ATOMS IN UNIT CELL: (SOUGHT)   1232; (FOUND)   1380
ATOMS IN ASYMMETRIC UNIT=   345
RO IN WFOURIER: MAX., SIGMA, MEAN: 0.13793E+06 0.87613E+04 0.32853E+00
MIN. INTERPEAK DISTANCE (A):   0.70

PEAK_NR	HEIGHT	X/A	Y/B	Z/C	MULT
1	1000	0.9025	0.7792	0.2252	1.0000
2	950	0.9332	0.6357	0.0871	1.0000
3	665	0.1542	0.9607	0.2164	1.0000
4	631	0.0772	0.6574	0.2198	1.0000
5	600	0.5005	0.6681	0.2461	1.0000
6	593	0.2253	0.4981	0.0264	1.0000
7	571	0.8995	0.6434	0.0360	1.0000
8	568	0.8007	0.4622	0.0528	1.0000
9	566	0.8358	0.1362	0.2399	1.0000
10	565	0.0185	0.1486	0.2404	1.0000
11	563	0.1028	0.0550	0.4524	1.0000
12	555	0.1443	0.2002	0.2349	1.0000
13	549	0.2025	0.0713	0.0608	1.0000
14	548	0.0451	0.0532	0.2178	1.0000
15	541	0.3106	0.0428	0.0391	1.0000
16	541	0.7790	0.0433	0.2039	1.0000
17	533	0.3252	0.9538	0.0060	1.0000
18	526	0.1284	0.0566	0.2239	1.0000
19	522	0.6813	0.4398	0.1002	1.0000
20	522	0.6846	0.2264	0.2041	1.0000
21	521	0.1019	0.5923	0.3095	1.0000
22	518	0.8075	0.5390	0.0890	1.0000
23	517	0.9242	0.9999	0.2027	1.0000
24	514	0.1781	0.9835	0.0030	1.0000
25	514	0.3657	0.1082	0.4738	1.0000
26	507	0.3950	0.0643	0.2426	1.0000
27	505	0.8607	0.1491	0.1498	1.0000
28	500	0.8866	0.3664	0.0182	1.0000
29	497	0.9533	0.2752	0.1369	1.0000

30	493	0.0437	0.5967	0.0367	1.0000
31	494	0.2996	0.1546	0.2440	1.0000
32	493	0.9294	0.4581	0.2902	1.0000
33	491	0.9465	0.3075	0.2231	1.0000
34	491	0.2436	0.0065	0.4488	1.0000
35	488	0.0104	0.6827	0.3143	1.0000
36	489	0.3106	0.8324	0.4080	1.0000
37	483	0.0345	0.6941	0.2542	1.0000
38	483	0.6188	0.1582	0.2216	1.0000
39	477	0.1061	0.1532	0.2347	1.0000
40	477	0.2244	0.2242	0.1975	1.0000
41	475	0.7648	0.2054	0.1333	1.0000
42	474	0.0559	0.6928	0.0395	1.0000
43	473	0.8854	0.2144	0.3065	1.0000
44	471	0.2362	0.6837	0.0389	1.0000
45	470	0.2847	0.6597	0.2127	1.0000
46	470	0.7557	0.3325	0.4444	1.0000
47	469	0.7002	0.5668	0.1118	1.0000
48	468	0.8122	0.2269	0.2403	1.0000
49	468	0.2006	0.8422	0.9982	1.0000
50	468	0.8932	0.4484	0.0029	1.0000
51	465	0.1295	0.7994	0.0348	1.0000
52	465	0.6681	0.5073	0.0488	1.0000
53	461	0.8025	0.5608	0.0523	1.0000
54	461	0.8474	0.3335	0.1842	1.0000
55	461	0.8682	0.2379	0.1897	1.0000
56	460	0.1662	0.1077	0.2312	1.0000
57	460	0.1761	0.9447	0.3862	1.0000
58	459	0.0950	0.6412	0.0366	1.0000
59	457	0.5717	0.4333	0.0170	1.0000
60	455	0.1317	0.6843	0.2592	1.0000
61	450	0.2082	0.5840	0.2039	1.0000
62	448	0.6051	0.4206	0.0357	1.0000
63	443	0.4121	0.8167	0.0037	1.0000
64	440	0.9430	0.5993	0.0397	1.0000
65	439	0.8471	0.1407	0.1876	1.0000
66	437	0.1803	0.5427	0.0303	1.0000
67	438	0.4343	0.6085	0.2260	1.0000
68	437	0.3690	0.8718	0.4606	1.0000
69	437	0.5622	0.3208	0.0720	1.0000
70	434	0.8673	0.0459	0.1993	1.0000
71	433	0.2097	0.9412	0.0589	1.0000
72	432	0.0382	0.5010	0.0269	1.0000
73	431	0.9764	0.3001	0.2053	1.0000

74	428	0.1926	0.6391	0.0371	1.0000
75	428	0.7160	0.2232	0.2401	1.0000
76	426	0.1174	0.6677	0.2005	1.0000
77	425	0.2785	0.0114	0.2282	1.0000
78	420	0.0808	0.4556	0.0211	1.0000
79	421	0.2539	0.1101	0.2372	1.0000
80	416	0.1769	0.4545	0.0213	1.0000
81	415	0.7059	0.4281	0.4391	1.0000
82	412	0.7847	0.5815	0.0758	1.0000
83	411	0.1224	0.3761	0.1681	1.0000
84	411	0.1900	0.0093	0.2226	1.0000
85	409	0.0844	0.5459	0.0307	1.0000
86	409	0.3407	0.1120	0.4927	1.0000
87	408	0.7725	0.3588	0.0964	1.0000
88	408	0.0321	0.7068	0.2288	1.0000
89	407	0.7535	0.4176	0.1037	1.0000
90	406	0.4329	0.6404	0.2961	1.0000
91	402	0.9295	0.4064	0.0134	1.0000
92	403	0.6077	0.4615	0.0516	1.0000
93	402	0.6463	0.3330	0.0730	1.0000
94	400	0.8011	0.4966	0.1227	1.0000
95	399	0.0121	0.0049	0.2105	1.0000
96	398	0.2770	0.6084	0.2127	1.0000
97	397	0.2247	0.4055	0.0164	1.0000
98	396	0.0293	0.4067	0.0156	1.0000
99	395	0.3558	0.9629	0.0671	1.0000
100	394	0.6744	0.2017	0.2206	1.0000
101	394	0.3147	0.0579	0.2355	1.0000
102	391	0.2056	0.8234	0.0358	1.0000
103	389	0.7951	0.1419	0.1666	1.0000
104	387	0.1750	0.3631	0.0111	1.0000
105	386	0.2988	0.0492	0.0015	1.0000
106	384	0.2685	0.9934	0.0038	1.0000
107	384	0.0784	0.3614	0.0116	1.0000
108	384	0.0688	0.3070	0.2001	1.0000
109	380	0.0917	0.6886	0.3117	1.0000
110	376	0.9601	0.4062	0.2966	1.0000
111	377	0.8506	0.1214	0.3095	1.0000
112	375	0.4449	0.6327	0.2438	1.0000
113	374	0.2574	0.8369	0.0167	1.0000

114	375	0.7662	0.5375	0.1077	1.0000
115	372	0.5520	0.9316	0.0198	1.0000
116	371	0.2725	0.0437	0.0580	1.0000
117	371	0.1362	0.9321	0.0070	1.0000
118	370	0.6959	0.2911	0.0258	1.0000
119	367	0.1223	0.3066	0.1451	1.0000
120	363	0.8373	0.1814	0.1333	1.0000
121	361	0.3272	0.7939	0.0102	1.0000
122	361	0.1896	0.5247	0.2509	1.0000
123	360	0.1031	0.4507	0.3047	1.0000
124	359	0.2767	0.0771	0.0224	1.0000
125	359	0.9992	0.1924	0.1254	1.0000
126	358	0.5888	0.0653	0.2276	1.0000
127	355	0.3260	0.0163	0.0745	1.0000
128	350	0.9132	0.2890	0.1872	1.0000
129	351	0.2527	0.8049	0.4550	1.0000
130	349	0.5513	0.0800	0.2080	1.0000
131	344	0.8947	0.3876	0.1804	1.0000
132	344	0.7091	0.2504	0.0160	1.0000
133	343	0.9442	0.7167	0.2216	1.0000
134	344	0.6178	0.1191	0.2379	1.0000
135	342	0.1163	0.2902	0.1833	1.0000
136	342	0.1954	0.5268	0.2024	1.0000
137	342	0.3044	0.8536	0.4455	1.0000
138	339	0.6251	0.3325	0.0216	1.0000
139	336	0.7770	0.3234	0.1679	1.0000
140	334	0.8239	0.6339	0.0810	1.0000
141	335	0.4880	0.9833	0.4420	1.0000
142	333	0.4826	0.1674	0.2003	1.0000
143	332	0.3538	0.5745	0.2209	1.0000
144	331	0.0113	0.2380	0.1340	1.0000
145	331	0.5178	0.0499	0.1949	1.0000
146	327	0.0501	0.4050	0.3027	1.0000
147	326	0.7634	0.0904	0.4661	1.0000
148	317	0.7129	0.7060	0.0152	1.0000
149	318	0.1212	0.3312	0.1662	1.0000
150	315	0.2724	0.0129	0.0954	1.0000
151	313	0.6725	0.2783	0.2467	1.0000
152	312	0.9022	0.1809	0.1178	1.0000
153	309	0.6397	0.3090	0.1088	1.0000
154	307	0.9679	0.4356	0.4919	1.0000
155	305	0.7293	0.4998	0.3301	1.0000
156	303	0.1203	0.0431	0.1648	1.0000
157	303	0.5081	0.5289	0.0737	1.0000
158	302	0.1773	0.4146	0.0813	1.0000
159	301	0.9315	0.5431	0.2240	1.0000
160	301	0.0156	0.3842	0.4881	1.0000
161	300	0.7338	0.9297	0.0284	1.0000
162	301	0.2072	0.2760	0.1905	1.0000
163	299	0.1128	0.2524	0.1415	1.0000
164	298	0.3035	0.7914	0.2309	1.0000
165	297	0.5290	0.3058	0.0217	1.0000
166	296	0.9001	0.4170	0.1203	1.0000
167	294	0.1670	0.9944	0.1625	1.0000
168	291	0.0452	0.5734	0.1337	1.0000
169	289	0.9283	0.2121	0.0277	1.0000
170	290	0.0281	0.5014	0.1585	1.0000
171	289	0.4503	0.9489	0.0683	1.0000
172	288	0.1933	0.8153	0.0726	1.0000
173	285	0.5947	0.9797	0.0251	1.0000
174	285	0.6938	0.3225	0.2346	1.0000
175	284	0.4535	0.4396	0.0650	1.0000
176	282	0.9343	0.6513	0.3771	1.0000
177	281	0.7989	0.4690	0.2222	1.0000
178	282	0.8656	0.4535	0.2292	1.0000
179	280	0.3011	0.0434	0.1765	1.0000
180	279	0.2236	0.3213	0.2080	1.0000
181	278	0.2559	0.5922	0.3397	1.0000
182	278	0.3961	0.3844	0.4817	1.0000
183	278	0.0081	0.9744	0.0242	1.0000
184	278	0.6855	0.3686	0.4434	1.0000
185	277	0.7484	0.4900	0.1414	1.0000
186	276	0.2841	0.2230	0.0958	1.0000
187	276	0.7761	0.5249	0.2139	1.0000
188	275	0.2212	0.2420	0.4967	1.0000
189	275	0.1283	0.3277	0.2169	1.0000
190	274	0.4948	0.6224	0.2090	1.0000
191	273	0.3188	0.1318	0.0254	1.0000
192	269	0.3215	0.4072	0.0175	1.0000
193	266	0.0685	0.5266	0.1421	1.0000
194	262	0.9637	0.5270	0.1677	1.0000
195	260	0.1059	0.3843	0.2263	1.0000
196	260	0.0065	0.1246	0.0413	1.0000
197	259	0.6652	0.0875	0.4675	1.0000
198	258	0.1594	0.0920	0.1723	1.0000
199	259	0.9034	0.3621	0.2961	1.0000
200	258	0.6270	0.7319	0.0206	1.0000
201	257	0.8741	0.1705	0.0322	1.0000
202	257	0.6635	0.4609	0.2730	1.0000
203	256	0.6865	0.9781	0.0302	1.0000

204	256	0.8734	0.2149	0.0976	1.0000
205	250	0.2494	0.0890	0.1762	1.0000
206	251	0.2882	0.3754	0.2718	1.0000
207	250	0.3073	0.9674	0.1087	1.0000
208	248	0.8490	0.5525	0.2171	1.0000
209	243	0.6936	0.8893	0.0220	1.0000
210	244	0.0038	0.4497	0.0744	1.0000
211	243	0.0720	0.0765	0.3378	1.0000
212	242	0.3961	0.1618	0.1453	1.0000
213	238	0.8515	0.7789	0.0613	1.0000
214	235	0.9383	0.4932	0.2302	1.0000
215	231	0.7706	0.7867	0.0717	1.0000
216	230	0.0500	0.1586	0.0389	1.0000
217	230	0.2396	0.2175	0.0790	1.0000
218	230	0.1941	0.6339	0.1694	1.0000
219	229	0.5749	0.8094	0.2249	1.0000
220	227	0.1250	0.3469	0.1281	1.0000
221	227	0.0605	0.4748	0.0818	1.0000
222	224	0.0232	0.2080	0.0313	1.0000
223	225	0.6547	0.7657	0.4019	1.0000
224	223	0.9766	0.5983	0.1441	1.0000
225	221	0.7968	0.1868	0.0870	1.0000
226	220	0.9193	0.1273	0.0383	1.0000
227	218	0.2739	0.0664	0.1052	1.0000
228	216	0.1487	0.4698	0.0845	1.0000
229	213	0.3569	0.2982	0.0813	1.0000
230	212	0.1788	0.6496	0.0919	1.0000
231	212	0.3700	0.2002	0.1477	1.0000
232	209	0.5165	0.4643	0.2189	1.0000
233	208	0.5394	0.1947	0.1448	1.0000
234	208	0.0514	0.7267	0.4176	1.0000
235	209	0.0851	0.3070	0.4386	1.0000
236	208	0.1804	0.6871	0.1064	1.0000
237	206	0.3484	0.4044	0.1426	1.0000
238	205	0.2467	0.6802	0.1224	1.0000
239	204	0.4752	0.6471	0.4143	1.0000
240	203	0.4719	0.4510	0.1260	1.0000
241	199	0.3840	0.5885	0.4323	1.0000
242	198	0.0060	0.0037	0.1085	1.0000
243	196	0.5716	0.1599	0.0517	1.0000
244	191	0.1045	0.3834	0.0710	1.0000
245	191	0.0266	0.3979	0.0691	1.0000
246	188	0.4776	0.4356	0.2298	1.0000
247	187	0.5267	0.1812	0.0676	1.0000
248	187	0.4210	0.8468	0.1561	1.0000
249	186	0.2295	0.6148	0.0916	1.0000
250	185	0.4321	0.5937	0.4132	1.0000
251	184	0.6153	0.1134	0.0516	1.0000
252	185	0.9978	0.0084	0.0939	1.0000
253	183	0.4827	0.7471	0.3482	1.0000
254	181	0.9336	0.9848	0.1268	1.0000
255	179	0.5471	0.4560	0.1973	1.0000
256	178	0.5228	0.4038	0.1893	1.0000
257	175	0.4920	0.1559	0.1428	1.0000
258	171	0.5748	0.9716	0.1276	1.0000
259	168	0.6777	0.8261	0.2337	1.0000
260	166	0.6475	0.9384	0.1591	1.0000
261	162	0.6835	0.7982	0.4352	1.0000
262	159	0.2736	0.7989	0.1626	1.0000
263	159	0.5717	0.9870	0.1523	1.0000
264	158	0.4018	0.4465	0.3782	1.0000
265	156	0.3234	0.4435	0.1499	1.0000
266	156	0.3103	0.1546	0.3241	1.0000
267	152	0.3038	0.6368	0.1215	1.0000
268	153	0.4838	0.4932	0.1344	1.0000
269	149	0.2600	0.2493	0.0622	1.0000
270	149	0.5776	0.8984	0.2130	1.0000
271	147	0.4195	0.4141	0.1249	1.0000
272	146	0.2534	0.3333	0.4243	1.0000
273	145	0.4732	0.8134	0.1584	1.0000
274	146	0.3808	0.7670	0.1718	1.0000
275	142	0.8863	0.0005	0.1207	1.0000
276	141	0.5474	0.8999	0.3052	1.0000
277	141	0.6553	0.2905	0.3261	1.0000
278	138	0.8365	0.8776	0.0794	1.0000
279	137	0.1517	0.5269	0.3973	1.0000
280	135	0.0016	0.7654	0.1492	1.0000
281	134	0.4044	0.2502	0.1490	1.0000
282	133	0.3292	0.8352	0.1623	1.0000
283	129	0.9086	0.8644	0.0693	1.0000
284	124	0.2938	0.6081	0.1037	1.0000
285	124	0.7217	0.7486	0.1784	1.0000
286	122	0.1327	0.5013	0.0256	1.0000
287	122	0.7528	0.7201	0.1488	1.0000
288	120	0.4206	0.7426	0.0674	1.0000
289	121	0.8073	0.6570	0.1029	1.0000
290	118	0.4074	0.7333	0.0820	1.0000
291	117	0.9524	0.7961	0.1201	1.0000
292	117	0.5206	0.2599	0.3319	1.0000
293	116	0.6233	0.3888	0.0185	1.0000

294	114	0.4435	0.7616	0.1008	1.0000
295	113	0.0315	0.2555	0.3714	1.0000
296	113	0.8859	0.7793	0.1186	1.0000
297	112	0.1283	0.2397	0.3479	1.0000
298	109	0.1417	0.5972	0.0356	1.0000
299	109	0.2812	0.2119	0.3168	1.0000
300	108	0.8988	0.0148	0.0860	1.0000
301	107	0.6952	0.6609	0.1534	1.0000
302	106	0.5004	0.7864	0.0618	1.0000
303	104	0.5210	0.8127	0.0717	1.0000
304	103	0.3769	0.4994	0.1533	1.0000
305	102	0.8480	0.5154	0.0509	1.0000
306	103	0.9601	0.8573	0.1487	1.0000
307	102	0.2731	0.8744	0.3327	1.0000
308	100	0.5088	0.7808	0.0878	1.0000
309	97	0.8291	0.8080	0.2275	1.0000
310	95	0.4950	0.7518	0.1010	1.0000
311	91	0.4944	0.6848	0.0726	1.0000
312	90	0.6486	0.6283	0.2257	1.0000
313	90	0.9644	0.1673	0.0396	1.0000
314	88	0.1920	0.0831	0.0219	1.0000
315	88	0.0646	0.8045	0.1491	1.0000
316	89	0.9150	0.1150	0.1476	1.0000
317	86	0.4544	0.7101	0.0632	1.0000
318	85	0.9925	0.6428	0.0376	1.0000
319	84	0.2679	0.7219	0.1327	1.0000
320	83	0.1503	0.6894	0.0365	1.0000
321	82	0.1226	0.8232	0.1329	1.0000
322	82	0.3969	0.1756	0.3690	1.0000
323	81	0.3405	0.7108	0.1124	1.0000
324	81	0.5211	0.1602	0.4375	1.0000
325	80	0.5504	0.7052	0.0974	1.0000
326	80	0.4450	0.2871	0.1539	1.0000
327	79	0.2312	0.2603	0.1653	1.0000
328	80	0.0740	0.1035	0.2284	1.0000
329	79	0.5809	0.6556	0.1803	1.0000
330	76	0.9025	0.5327	0.0874	1.0000
331	75	0.3174	0.7055	0.0929	1.0000
332	74	0.5142	0.2225	0.1669	1.0000
333	74	0.2745	0.1839	0.1859	1.0000
334	74	0.8786	0.1642	0.2090	1.0000
335	74	0.1042	0.8488	0.0695	1.0000
336	73	0.8989	0.7782	0.1364	1.0000
337	74	0.5694	0.6389	0.2067	1.0000
338	72	0.5752	0.7383	0.0881	1.0000
339	71	0.1478	0.0329	0.0313	1.0000
340	70	0.8512	0.9745	0.0185	1.0000
341	71	0.5069	0.0412	0.1502	1.0000
342	70	0.6458	0.6968	0.1687	1.0000
343	70	0.3187	0.2287	0.1894	1.0000
344	70	0.7964	0.5310	0.1903	1.0000
345	70	0.3473	0.3298	0.1124	1.0000

```
COPY OF FILE.CDR:
  ==== == =======

ACTINOMICIN  P212121    8 pdb_sf 1A7Z
CELL
14.803 24.780 65.059 90.00 90.00 90.00
LATTICE
P
SYMMETRY
X,Y,Z
1/2-X,-Y,1/2+Z
-X,1/2+Y,1/2-Z
1/2+X,1/2-Y,-Z
CONTENTS
CL   C       H
8   1224    400
```

```
&CONTROL
IHKL=1,DSFOU=0.90,NSET=25,NCYCLE=29,RVMIN=100.,binit=0.5769230,
IATOMS=1,FROLW=-2.5,FSIGip=3.7,DELTLV=1.1,NITER=3,IFOUSG=1,
IBREAK=0,IPHASE=2,itest=1,iexcc=0/


++++++++++++++++++++++++++++++++++++++++++++++++++++++++++++++
   Job finished on 19- 2-2024   at 19:39:38
   Elapsed time:       107 seconds
++++++++++++++++++++++++++++++++++++++++++++++++++++++++++++++
```

```
  XLENS_SMARV241: A DELTA DIRECT-METHODS PROGRAM
  Crystal structure solution with the SMAR phasing algorithm
  Copyright Prof. Jordi Rius (Palleiro)
  Rius J. Acta Cryst (2020) A76 489-493
  Institut de Ciencia de Materials de Barcelona (CSIC)
  Date is 19- 2-2024   at 19:35: 9
============================================================


    TITL ALFA1

   Crystal data:
    a =   20.846 =     alpha=  102.400
    b =   20.909 =     beta=    95.330
    c =   27.057 =     gamma=  119.620

    Reciprocal lattice constants (a, b, c):
    (sinth/l)2=  0.000806*H2 + 0.000832*K2 + 0.000379*L2 +
                0.000866*HK + 0.000259*HL + 0.000338*KL

    Unit cell volume (a,b,c) =      9734.04

    Unit cell (a,b,c) is P centered
     nodes at:
      1)  0.000000       0.000000       0.000000

     Symmetry operations:
     R11  R12  R13     R21  R22  R23     R31  R32  R33    T1     T2     T3
 1)    1    0    0       0    1    0       0    0    1    0.00   0.00   0.00

         Unit cell contents
     Symbol  Atomic_number    Number in cell    type    scat_power
       CL         17               1            1         17.00
       O           8             110            2          8.00
       N           7              65            3          7.00
       C           6             328            4          6.00
       H           1             500            5          1.00

    X-RAYS

    RANDOM starting phases
```

```
-----------------------------------------------------------------

     Intensity data information:
     ===========================

     Input data are F**2
     DSMIN (Angs.):            0.9041
     DSFOU (Angs.):            0.9041
-----------------------------------------------------------------

     FORBIDDEN REFLECTIONS IN DATA FILE *.HKL:
     ===========================================

    H    K    L        XO       DXO      NEQ
-----------------------------------------------------------------

     Rsigma(I) of equivalent reflections
     Rsig:    0.00000  Nref:         0

     ds_interval        Rsigma       Nref

     N. UNIQUE REFLECTIONS (measured,theory):       23681       27574


     INFORMATION OF DATA IN P1:
     MEAS. INTENSITIES (OBS+UNOBS)IN XOHEM.HKL=        23681
     NOT MEASURED INTENSITIES in XOUNMEAS.HKL=        3893
     FRACTION MEASURED INTENSITIES >DSFOU:     0.859
     HMAX=   23  KMAX=   23  LMAX=   29


-----------------------------------------------------------------

     SCALING OF MEASURED INTENSITIES:
     =================================


      SCALE FACTOR:        0.9450  F(EXP)/SK = F(ABS)
      B OVERALL   :        4.1645
      R(Wilson)   :       26.0665
      DSFOU       :        0.9041
      FACDF       :        2.0000  Observed if XO > FACDF*sig(XO)

      SHELL  DMEAN  DINF  SFC2/SFO2  Nref  f.unobs
```

1	1.95	1.55	1.00740	5539	0.0052
2	1.35	1.23	1.18017	5471	0.0238
3	1.14	1.07	0.81678	5323	0.0541
4	1.02	0.97	0.84447	4402	0.1197
5	0.94	0.90	1.21945	2946	0.2980

DATA NORMALIZING:
================

NUMBER OF INPUT REFLEXIONS = 23681
F(ABS) = F(EXP) / 0.9450

	CENT	ACENT	HK0	0KL	H0L	REST
</E2-1/>	0.968	0.736	0.866	0.810	0.726	0.771

E2MEAN : 0.966802

<E**2> ACCORDING TO PARITY GROUPS (D-SPACING > 0.904

	ALL	GGG	GGU	GUG	GUU	UGG	UGU	UUG	UUU
E^2	1.000	1.051	1.105	1.017	1.032	0.966	0.968	0.955	0.906
N	23681	2975	2952	2945	2954	2952	2973	2958	2972

PHASE REFINEMENT DETAILS:
========================

TOTAL N. OF E-VALUES = 23681
D-SPACING CUT-OFF = 0.90
<E>, <E2> = 0.8711 1.0000

N. SETS AND CYCLES = 25 42
ELIM FOR K REFLECTIONS = 0.00
N.REFLECTIONS > EKLIM = 23681
ELIM FOR H REFLECTIONS = 1.00
N.REFLECTIONS > EHLIM = 8173
N. ATOMS IN UNIT_CELL (NCELL) = 504
PIXELS(0); P_ATOMS(1); OMIT(2) = 1
FRAC. RANDOMLY DELETED ATOMS = 0.000

GRID SPACING IN ANGS. = 0.33
N.GRID POINTS (XYZ) = 64 64 80
LOWER SDV FACTOR FOR MASK = -2.50
ESD FACT FOR PEAK ACCEPTANCE (ATOMS=1,2) = 3.70

ESD FACTOR IN SEARCH (DIF.FOURIER) = 1.10

ITER	SRO2	SDEL2	2S_RDM	P	Q	RDEL	%0	%-1	CCro’	CCro”
1	0.114E+11	0.197E+11	0.0129	0.5187	0.8657	1.3972	49.897	0.181	-0.0096	0.5745
2	0.115E+11	0.197E+11	-0.4335	0.5704	0.8545	0.9914	51.595	0.083	0.3104	0.6865
3	0.115E+11	0.197E+11	-0.5080	0.5796	0.8515	0.9232	51.923	0.080	0.3615	0.6807
4	0.115E+11	0.197E+11	-0.5241	0.5826	0.8516	0.9101	52.008	0.083	0.3720	0.7005
5	0.115E+11	0.197E+11	-0.5599	0.5867	0.8613	0.8882	52.110	0.079	0.3938	0.7065
6	0.115E+11	0.197E+11	-0.5523	0.5881	0.8585	0.8943	52.040	0.092	0.3886	0.6836
7	0.115E+11	0.197E+11	-0.5739	0.5897	0.8640	0.8798	52.226	0.084	0.4020	0.7216
8	0.115E+11	0.197E+11	-0.5968	0.5924	0.8564	0.8520	52.289	0.071	0.4189	0.7189
9	0.115E+11	0.197E+11	-0.5944	0.5932	0.8602	0.8590	52.243	0.072	0.4160	0.7098
10	0.115E+11	0.197E+11	-0.6125	0.5967	0.8607	0.8449	52.344	0.086	0.4274	0.7061
11	0.115E+11	0.197E+11	-0.6188	0.5981	0.8613	0.8406	52.328	0.078	0.4311	0.7198
12	0.115E+11	0.197E+11	-0.6349	0.6013	0.8599	0.8263	52.478	0.094	0.4415	0.7078
13	0.115E+11	0.197E+11	-0.6194	0.5985	0.8621	0.8412	52.318	0.082	0.4312	0.7157
14	0.115E+11	0.197E+11	-0.6155	0.6000	0.8676	0.8520	52.303	0.091	0.4266	0.7046
15	0.115E+11	0.197E+11	-0.6128	0.5993	0.8599	0.8465	52.390	0.086	0.4268	0.7108
16	0.115E+11	0.197E+11	-0.6388	0.5994	0.8595	0.8201	52.455	0.075	0.4450	0.7128
17	0.115E+11	0.197E+11	-0.6388	0.5998	0.8582	0.8192	52.442	0.076	0.4452	0.7232
18	0.115E+11	0.197E+11	-0.6459	0.6033	0.8631	0.8204	52.487	0.089	0.4476	0.7338
19	0.115E+11	0.197E+11	-0.6453	0.6024	0.8631	0.8202	52.379	0.085	0.4475	0.7268
20	0.115E+11	0.197E+11	-0.6432	0.6048	0.8607	0.8223	52.344	0.104	0.4457	0.7114
21	0.115E+11	0.197E+11	-0.6512	0.6050	0.8612	0.8150	52.401	0.094	0.4511	0.7109
22	0.115E+11	0.197E+11	-0.6472	0.6027	0.8663	0.8218	52.294	0.088	0.4478	0.7186
23	0.115E+11	0.197E+11	-0.6302	0.6045	0.8645	0.8388	52.358	0.107	0.4359	0.7069
24	0.115E+11	0.197E+11	-0.6326	0.6031	0.8646	0.8351	52.214	0.102	0.4380	0.7118
25	0.115E+11	0.197E+11	-0.6304	0.6047	0.8640	0.8383	52.240	0.111	0.4361	0.7218
26	0.115E+11	0.197E+11	-0.6397	0.6044	0.8622	0.8269	52.415	0.094	0.4431	0.7093
27	0.115E+11	0.197E+11	-0.6358	0.6061	0.8684	0.8387	52.297	0.105	0.4382	0.7036
28	0.115E+11	0.197E+11	-0.6488	0.6049	0.8663	0.8224	52.313	0.093	0.4482	0.7233
29	0.115E+11	0.197E+11	-0.6568	0.6073	0.8726	0.8232	52.337	0.104	0.4511	0.7182
30	0.115E+11	0.197E+11	-0.6692	0.6083	0.8675	0.8067	52.439	0.092	0.4606	0.7309
31	0.115E+11	0.197E+11	-0.6481	0.6091	0.8699	0.8308	52.237	0.113	0.4452	0.6985
32	0.115E+11	0.197E+11	-0.6600	0.6096	0.8712	0.8209	52.369	0.099	0.4528	0.7126
33	0.115E+11	0.197E+11	-0.6556	0.6113	0.8743	0.8301	52.268	0.123	0.4484	0.6922
34	0.115E+11	0.197E+11	-0.6739	0.6094	0.8715	0.8070	52.430	0.094	0.4624	0.7236
35	0.115E+11	0.197E+11	-0.6656	0.6102	0.8722	0.8168	52.354	0.099	0.4562	0.7167
36	0.115E+11	0.197E+11	-0.6844	0.6122	0.8780	0.8059	52.365	0.100	0.4667	0.7413
37	0.115E+11	0.197E+11	-0.7261	0.6215	0.8826	0.7780	52.605	0.092	0.4902	0.7552
38	0.115E+11	0.197E+11	-0.8590	0.6416	0.8927	0.6754	53.130	0.072	0.5675	0.8185
39	0.115E+11	0.197E+11	-1.0481	0.6785	0.8816	0.5120	53.882	0.060	0.6776	0.8857
40	0.115E+11	0.197E+11	-1.0824	0.6855	0.8736	0.4767	54.148	0.056	0.6994	0.9054

41	0.115E+11	0.197E+11	-1.0892	0.6859	0.8708	0.4675	54.145	0.049	0.7047	0.9087
42	0.115E+11	0.197E+11	-1.0889	0.6868	0.8745	0.4723	54.183	0.055	0.7026	0.9056

SUMMARY OF SOLUTIONS:

SET	CCH	SEED	CCK	NCYC	NITER	RVAL(ini,end)		PKS(ini,end)		RO/SIG
1	0.9240	0.6923080	0.8227	42	3	19.0	11.6	603	574	25.23

TOTAL NUMBER OF CYCLES IS 42 IEXCC = 0

PEAK SEARCH:
===========

SOLUTION N. 1 R = 11.603117 ITERATIONS= 3

NUMBER OF E AND CUT-OFF VALUE 13697 0.70
GRID: NX= 64 NY= 64 NZ= 80 SIZE(ANGS)= 0.33
ATOMS IN UNIT CELL: (SOUGHT) 504; (FOUND) 575
ATOMS IN ASYMMETRIC UNIT= 574
RO IN WFOURIER: MAX., SIGMA, MEAN: 0.79139E+05 0.41346E+04 -0.26649E-04
MIN. INTERPEAK DISTANCE (A): 0.70

PEAK_NR	HEIGHT	X/A	Y/B	Z/C	MULT
1	1000	0.0260	0.0524	0.9151	1.0000
2	577	0.2969	0.8426	0.1523	1.0000
3	545	0.4420	0.9378	0.2631	1.0000
4	525	0.4399	0.5570	0.8986	1.0000
5	524	0.0464	0.3820	0.7235	1.0000
6	523	0.8306	0.5185	0.2716	1.0000
7	522	0.5361	0.9011	0.1026	1.0000
8	503	0.1106	0.0793	0.7322	1.0000
9	502	0.8594	0.8463	0.6348	1.0000
10	499	0.9726	0.9191	0.7575	1.0000
11	496	0.3040	0.0750	0.2497	1.0000
12	493	0.2485	0.3718	0.6400	1.0000
13	493	0.2052	0.5637	0.7458	1.0000
14	492	0.4968	0.4371	0.2620	1.0000
15	481	0.9761	0.5608	0.6870	1.0000
16	479	0.1327	0.9062	0.2471	1.0000
17	479	0.4037	0.9381	0.6373	1.0000
18	477	0.0491	0.9185	0.1434	1.0000
19	478	0.5487	0.6411	0.8839	1.0000
20	477	0.5262	0.7483	0.9406	1.0000

21	477	0.7042	0.9028	0.0185	1.0000
22	476	0.2986	0.6402	0.0635	1.0000
23	471	0.1380	0.9838	0.6338	1.0000
24	471	0.5684	0.0602	0.2028	1.0000
25	470	0.0775	0.0966	0.2092	1.0000
26	470	0.1578	0.3148	0.2131	1.0000
27	467	0.8588	0.0113	0.6387	1.0000
28	467	0.3794	0.4714	0.7466	1.0000
29	464	0.3027	0.0163	0.7884	1.0000
30	462	0.8923	0.4878	0.7827	1.0000
31	461	0.6662	0.3485	0.2088	1.0000
32	460	0.1108	0.3786	0.6622	1.0000
33	457	0.4427	0.8613	0.1490	1.0000
34	457	0.4876	0.4069	0.1233	1.0000
35	454	0.1061	0.9234	0.7412	1.0000
36	451	0.7642	0.0045	0.2526	1.0000
37	449	0.9235	0.9970	0.7495	1.0000
38	447	0.8601	0.4254	0.2586	1.0000
39	447	0.6306	0.8275	0.6827	1.0000
40	448	0.2447	0.0668	0.7481	1.0000
41	446	0.2336	0.5228	0.6265	1.0000
42	446	0.7870	0.8543	0.6890	1.0000
43	445	0.3693	0.3352	0.1353	1.0000
44	446	0.2032	0.0686	0.1975	1.0000
45	446	0.9693	0.3801	0.2600	1.0000
46	446	0.7307	0.9097	0.6131	1.0000
47	445	0.7966	0.0117	0.7454	1.0000
48	445	0.1300	0.2819	0.0114	1.0000
49	445	0.5953	0.9369	0.0186	1.0000
50	445	0.9518	0.0218	0.1627	1.0000
51	445	0.9969	0.5386	0.2371	1.0000
52	445	0.3539	0.5773	0.6868	1.0000
53	444	0.3065	0.0040	0.1299	1.0000
54	444	0.8177	0.0086	0.1304	1.0000
55	442	0.4576	0.0088	0.1591	1.0000
56	436	0.4049	0.0994	0.6986	1.0000
57	437	0.7130	0.5796	0.7687	1.0000
58	435	0.6329	0.4247	0.1107	1.0000
59	436	0.9198	0.5442	0.1773	1.0000
60	436	0.1582	0.8631	0.6946	1.0000
61	434	0.7136	0.8044	0.0122	1.0000
62	433	0.4239	0.3402	0.0212	1.0000
63	432	0.5756	0.5662	0.7320	1.0000
64	431	0.2522	0.8892	0.2160	1.0000

65	430	0.1498	0.4412	0.1527	1.0000
66	430	0.6886	0.0256	0.2029	1.0000
67	430	0.4684	0.9097	0.7626	1.0000
68	430	0.1862	0.1918	0.9846	1.0000
69	430	0.6897	0.9647	0.8789	1.0000
70	428	0.6911	0.9211	0.1210	1.0000
71	429	0.2610	0.6407	0.9125	1.0000
72	427	0.4344	0.4259	0.6943	1.0000
73	427	0.2482	0.4831	0.7436	1.0000
74	427	0.7009	0.4710	0.6410	1.0000
75	427	0.5919	0.9051	0.7260	1.0000
76	427	0.4486	0.9092	0.9402	1.0000
77	426	0.0616	0.5474	0.7354	1.0000
78	424	0.5875	0.8349	0.1916	1.0000
79	424	0.3167	0.9058	0.6864	1.0000
80	423	0.1851	0.9282	0.1382	1.0000
81	423	0.1212	0.0171	0.2711	1.0000
82	422	0.1464	0.5468	0.2620	1.0000
83	420	0.0119	0.4401	0.1530	1.0000
84	419	0.9558	0.3922	0.6189	1.0000
85	417	0.9705	0.9411	0.0112	1.0000
86	416	0.5455	0.3797	0.2088	1.0000
87	417	0.4099	0.0254	0.2525	1.0000
88	413	0.0162	0.8969	0.6438	1.0000
89	413	0.3624	0.6113	0.1159	1.0000
90	412	0.1606	0.5132	0.8921	1.0000
91	408	0.1661	0.4577	0.2772	1.0000
92	407	0.4817	0.0140	0.0047	1.0000
93	408	0.5672	0.9201	0.2391	1.0000
94	405	0.2879	0.0107	0.6591	1.0000
95	403	0.7492	0.4973	0.1630	1.0000
96	403	0.6884	0.5148	0.2504	1.0000
97	401	0.2447	0.7384	0.0512	1.0000
98	402	0.5584	0.8735	0.9043	1.0000
99	400	0.1097	0.3753	0.9960	1.0000
100	401	0.5046	0.5860	0.6799	1.0000
101	399	0.0298	0.3408	0.2059	1.0000
102	399	0.9207	0.4078	0.7300	1.0000
103	399	0.0278	0.9031	0.9136	1.0000

104	399	0.0443	0.8424	0.6508	1.0000
105	398	0.8240	0.5303	0.6922	1.0000
106	395	0.4818	0.0827	0.2479	1.0000
107	392	0.7522	0.3636	0.1608	1.0000
108	393	0.3894	0.9565	0.2587	1.0000
109	393	0.3116	0.2440	0.9363	1.0000
110	392	0.0048	0.0280	0.6712	1.0000
111	392	0.3461	0.4083	0.9114	1.0000
112	390	0.0010	0.9355	0.1617	1.0000
113	389	0.4022	0.4361	0.0140	1.0000
114	388	0.7651	0.9171	0.9378	1.0000
115	387	0.7021	0.7405	0.9011	1.0000
116	385	0.4855	0.1555	0.2488	1.0000
117	384	0.7805	0.3620	0.2517	1.0000
118	382	0.2504	0.9980	0.1405	1.0000
119	383	0.8865	0.3594	0.1430	1.0000
120	383	0.2500	0.3425	0.1672	1.0000
121	383	0.3760	0.6302	0.9578	1.0000
122	382	0.9692	0.9137	0.1979	1.0000
123	380	0.4656	0.9714	0.1108	1.0000
124	378	0.0793	0.4851	0.6363	1.0000
125	378	0.2476	0.8629	0.1653	1.0000
126	377	0.8280	0.3998	0.6497	1.0000
127	377	0.5041	0.0461	0.2002	1.0000
128	376	0.7073	0.0089	0.6872	1.0000
129	376	0.1974	0.0695	0.2874	1.0000
130	375	0.7598	0.5014	0.7783	1.0000
131	374	0.1748	0.9640	0.7880	1.0000
132	374	0.7255	0.3275	0.6839	1.0000
133	372	0.3970	0.5576	0.0296	1.0000
134	373	0.3586	0.4536	0.6192	1.0000
135	372	0.3300	0.6081	0.7254	1.0000
136	370	0.1608	0.9192	0.9023	1.0000
137	369	0.2620	0.4904	0.2082	1.0000
138	368	0.4862	0.2624	0.1378	1.0000
139	363	0.4362	0.3503	0.1395	1.0000
140	364	0.8251	0.2191	0.2887	1.0000
141	363	0.6555	0.4695	0.3261	1.0000
142	363	0.3449	0.9365	0.6494	1.0000
143	362	0.0601	0.7660	0.0712	1.0000
144	362	0.8118	0.2855	0.2990	1.0000
145	360	0.1964	0.1442	0.1128	1.0000
146	360	0.7994	0.8452	0.6391	1.0000
147	360	0.9744	0.7630	0.6510	1.0000
148	360	0.1886	0.5796	0.8879	1.0000

149	360	0.8735	0.4702	0.0031	1.0000
150	359	0.0287	0.3781	0.2509	1.0000
151	360	0.4852	0.2017	0.3013	1.0000
152	359	0.3140	0.3917	0.6384	1.0000
153	358	0.1906	0.8567	0.1252	1.0000
154	359	0.4219	0.3085	0.7897	1.0000
155	358	0.2380	0.0637	0.1525	1.0000
156	357	0.9676	0.5585	0.2797	1.0000
157	357	0.3099	0.9722	0.6226	1.0000
158	357	0.6404	0.5746	0.2120	1.0000
159	356	0.3147	0.1199	0.7359	1.0000
160	356	0.7320	0.0498	0.1683	1.0000
161	354	0.9176	0.7193	0.6012	1.0000
162	355	0.0624	0.5451	0.6422	1.0000
163	353	0.1562	0.5200	0.9946	1.0000
164	353	0.4481	0.4838	0.7356	1.0000
165	353	0.4853	0.9118	0.1214	1.0000
166	351	0.3899	0.9254	0.0634	1.0000
167	352	0.4254	0.7068	0.9860	1.0000
168	349	0.5169	0.5379	0.7622	1.0000
169	350	0.7635	0.5595	0.7609	1.0000
170	350	0.4349	0.9991	0.7790	1.0000
171	349	0.1540	0.3593	0.0227	1.0000
172	348	0.6447	0.9674	0.2731	1.0000
173	348	0.0306	0.4154	0.6267	1.0000
174	348	0.5259	0.7837	0.8205	1.0000
175	347	0.7377	0.5572	0.1764	1.0000
176	348	0.0277	0.5455	0.7804	1.0000
177	345	0.0131	0.5758	0.3339	1.0000
178	346	0.9979	0.0644	0.7629	1.0000
179	344	0.7973	0.8524	0.7794	1.0000
180	343	0.9742	0.5271	0.1887	1.0000
181	343	0.1079	0.3973	0.7117	1.0000
182	344	0.5457	0.5211	0.8082	1.0000
183	344	0.5000	0.8253	0.8527	1.0000
184	342	0.2172	0.1726	0.0293	1.0000
185	343	0.0450	0.0606	0.7224	1.0000
186	342	0.8334	0.8576	0.7324	1.0000
187	342	0.9380	0.4753	0.7618	1.0000
188	340	0.2175	0.3753	0.1350	1.0000
189	341	0.7468	0.9917	0.1379	1.0000
190	340	0.9690	0.9672	0.1294	1.0000
191	340	0.3027	0.5263	0.6375	1.0000
192	339	0.9573	0.8271	0.9835	1.0000
193	338	0.4656	0.8126	0.1652	1.0000

194	339	0.1462	0.5939	0.8481	1.0000
195	337	0.7808	0.2926	0.2506	1.0000
196	337	0.3545	0.2965	0.8486	1.0000
197	337	0.5483	0.8588	0.2008	1.0000
198	337	0.7034	0.0007	0.2406	1.0000
199	336	0.3289	0.7029	0.1540	1.0000
200	336	0.9077	0.9205	0.7433	1.0000
201	334	0.3934	0.6545	0.7755	1.0000
202	334	0.4112	0.7615	0.1898	1.0000
203	333	0.0754	0.2646	0.5585	1.0000
204	334	0.2438	0.0188	0.7742	1.0000
205	333	0.5567	0.4376	0.2501	1.0000
206	333	0.2984	0.1716	0.7139	1.0000
207	332	0.2023	0.0752	0.1099	1.0000
208	330	0.7845	0.9853	0.6357	1.0000
209	331	0.1080	0.8797	0.6988	1.0000
210	330	0.1202	0.7951	0.1228	1.0000
211	329	0.8845	0.5038	0.2709	1.0000
212	328	0.2199	0.8438	0.3157	1.0000
213	327	0.1612	0.0094	0.8336	1.0000
214	327	0.4897	0.5653	0.8761	1.0000
215	327	0.4402	0.9715	0.8718	1.0000
216	324	0.7334	0.3626	0.2066	1.0000
217	324	0.2404	0.8991	0.5781	1.0000
218	324	0.8169	0.5865	0.7267	1.0000
219	324	0.7654	0.2804	0.3404	1.0000
220	323	0.0638	0.3287	0.5693	1.0000
221	323	0.3607	0.3006	0.6213	1.0000
222	323	0.0627	0.9605	0.6349	1.0000
223	323	0.2565	0.5455	0.7369	1.0000
224	323	0.0685	0.5286	0.8230	1.0000
225	321	0.4113	0.4984	0.2352	1.0000
226	322	0.1382	0.6281	0.6450	1.0000
227	321	0.4216	0.3972	0.0374	1.0000
228	321	0.2352	0.0663	0.2414	1.0000
229	321	0.3300	0.4934	0.5983	1.0000
230	320	0.7136	0.0941	0.1369	1.0000
231	320	0.3561	0.3623	0.6626	1.0000
232	320	0.9964	0.1386	0.7687	1.0000
233	318	0.9830	0.1999	0.1182	1.0000
234	318	0.0063	0.0786	0.1987	1.0000
235	319	0.9791	0.2653	0.9244	1.0000
236	317	0.6706	0.9650	0.7625	1.0000
237	318	0.4973	0.5049	0.8473	1.0000
238	317	0.1872	0.4350	0.7578	1.0000

239	316	0.9856	0.6885	0.0619	1.0000
240	316	0.9691	0.3105	0.1627	1.0000
241	316	0.0526	0.3531	0.6231	1.0000
242	315	0.7935	0.3545	0.6812	1.0000
243	316	0.9658	0.7824	0.8380	1.0000
244	315	0.0016	0.5023	0.1456	1.0000
245	315	0.1037	0.4243	0.2989	1.0000
246	314	0.0378	0.0097	0.6268	1.0000
247	314	0.7211	0.9867	0.7267	1.0000
248	312	0.9540	0.3716	0.1530	1.0000
249	312	0.3101	0.8940	0.2554	1.0000
250	312	0.1882	0.5187	0.2606	1.0000
251	312	0.8957	0.6519	0.7612	1.0000
252	313	0.7784	0.2144	0.7791	1.0000
253	312	0.3243	0.7649	0.8779	1.0000
254	311	0.6475	0.0249	0.3164	1.0000
255	310	0.2026	0.9246	0.5391	1.0000
256	309	0.8425	0.6580	0.6093	1.0000
257	309	0.5791	0.8412	0.6918	1.0000
258	309	0.6828	0.5494	0.2124	1.0000
259	308	0.0925	0.9379	0.2499	1.0000
260	308	0.2226	0.1501	0.3213	1.0000
261	308	0.6883	0.7770	0.5736	1.0000
262	308	0.6900	0.7638	0.8143	1.0000
263	308	0.8891	0.2773	0.8290	1.0000
264	308	0.2692	0.3890	0.9092	1.0000
265	307	0.0029	0.8764	0.2274	1.0000
266	307	0.7340	0.5286	0.3637	1.0000
267	307	0.8116	0.4452	0.5733	1.0000
268	308	0.7489	0.8492	0.5939	1.0000
269	306	0.1670	0.1540	0.0664	1.0000
270	306	0.1389	0.8579	0.5024	1.0000
271	305	0.2952	0.2329	0.7600	1.0000
272	305	0.3355	0.6510	0.1111	1.0000
273	304	0.6885	0.4235	0.1284	1.0000
274	305	0.7631	0.4733	0.6505	1.0000
275	303	0.6722	0.0254	0.7996	1.0000
276	304	0.2214	0.8109	0.8780	1.0000
277	303	0.7463	0.1720	0.0739	1.0000
278	303	0.7075	0.3569	0.1179	1.0000
279	302	0.2633	0.2950	0.2388	1.0000
280	302	0.2913	0.9133	0.3112	1.0000
281	302	0.9694	0.5816	0.3781	1.0000
282	303	0.7334	0.7492	0.8541	1.0000
283	302	0.8437	0.2708	0.6960	1.0000

284	300	0.5958	0.0028	0.3526	1.0000
285	301	0.3401	0.0817	0.6965	1.0000
286	299	0.8585	0.5938	0.0029	1.0000
287	300	0.7966	0.6443	0.0740	1.0000
288	299	0.8455	0.3414	0.7166	1.0000
289	298	0.2658	0.4041	0.0972	1.0000
290	299	0.7609	0.1253	0.0978	1.0000
291	299	0.8228	0.3940	0.5342	1.0000
292	299	0.7806	0.4169	0.6187	1.0000
293	298	0.8996	0.8496	0.0506	1.0000
294	297	0.4438	0.8542	0.5642	1.0000
295	295	0.6961	0.8605	0.0922	1.0000
296	295	0.2186	0.3179	0.2020	1.0000
297	294	0.0280	0.5523	0.6889	1.0000
298	295	0.8915	0.7230	0.7946	1.0000
299	293	0.8924	0.9051	0.0899	1.0000
300	292	0.3589	0.2790	0.7992	1.0000
301	291	0.4244	0.9300	0.7553	1.0000
302	291	0.2776	0.9729	0.9014	1.0000
303	290	0.9763	0.2627	0.1100	1.0000
304	290	0.2630	0.9744	0.5106	1.0000
305	291	0.9148	0.0323	0.9920	1.0000
306	290	0.4716	0.3156	0.1718	1.0000
307	290	0.9494	0.6832	0.5625	1.0000
308	289	0.6475	0.0757	0.9963	1.0000
309	288	0.2078	0.4327	0.1668	1.0000
310	288	0.9957	0.1429	0.2355	1.0000
311	289	0.9742	0.9710	0.5744	1.0000
312	289	0.7525	0.0336	0.6507	1.0000
313	289	0.7877	0.5552	0.8862	1.0000
314	285	0.9651	0.8647	0.2750	1.0000
315	284	0.9343	0.6629	0.0105	1.0000
316	285	0.5792	0.8678	0.5602	1.0000
317	284	0.7253	0.7780	0.7711	1.0000
318	284	0.0128	0.8418	0.3156	1.0000
319	284	0.9642	0.1578	0.8130	1.0000
320	284	0.8622	0.2341	0.9789	1.0000
321	283	0.8163	0.3461	0.9957	1.0000
322	283	0.7370	0.0745	0.8466	1.0000
323	282	0.7607	0.0901	0.9393	1.0000
324	282	0.4952	0.7839	0.6542	1.0000
325	281	0.5607	0.5805	0.6885	1.0000
326	279	0.7772	0.0954	0.5749	1.0000
327	279	0.2856	0.2355	0.5848	1.0000
328	279	0.4015	0.8931	0.0144	1.0000
329	279	0.6657	0.1395	0.0436	1.0000
330	279	0.2582	0.5529	0.2393	1.0000
331	278	0.1186	0.5620	0.1017	1.0000
332	276	0.4459	0.9443	0.9851	1.0000
333	274	0.8153	0.6353	0.2008	1.0000
334	274	0.4590	0.7172	0.7672	1.0000
335	275	0.4238	0.0936	0.9619	1.0000
336	274	0.9509	0.8759	0.0131	1.0000
337	273	0.7247	0.0303	0.8879	1.0000
338	271	0.6335	0.5017	0.2844	1.0000
339	272	0.1856	0.3581	0.7641	1.0000
340	270	0.0837	0.5735	0.1482	1.0000
341	270	0.6239	0.6064	0.6599	1.0000
342	269	0.3698	0.5372	0.5639	1.0000
343	269	0.5088	0.7390	0.9885	1.0000
344	269	0.2251	0.4155	0.0558	1.0000
345	265	0.1067	0.5442	0.0017	1.0000
346	265	0.4683	0.4489	0.0964	1.0000
347	264	0.9682	0.8544	0.8650	1.0000
348	264	0.8830	0.2572	0.7380	1.0000
349	261	0.7272	0.0625	0.6118	1.0000
350	260	0.7312	0.3482	0.0679	1.0000
351	260	0.6070	0.0788	0.3935	1.0000
352	260	0.5748	0.0295	0.6746	1.0000
353	261	0.5030	0.0185	0.8651	1.0000
354	259	0.1986	0.7658	0.7859	1.0000
355	259	0.2214	0.0426	0.8852	1.0000
356	257	0.1075	0.3709	0.3285	1.0000
357	257	0.0961	0.2502	0.5072	1.0000
358	254	0.2136	0.9764	0.9004	1.0000
359	254	0.0156	0.7751	0.2898	1.0000
360	254	0.8458	0.2496	0.7875	1.0000
361	253	0.5691	0.2446	0.3404	1.0000
362	252	0.9964	0.9429	0.5270	1.0000
363	251	0.7929	0.4250	0.0643	1.0000
364	252	0.4189	0.2039	0.0943	1.0000
365	252	0.2166	0.8729	0.3733	1.0000
366	251	0.5713	0.0473	0.8924	1.0000
367	251	0.7094	0.8555	0.0349	1.0000
368	251	0.5512	0.3923	0.6524	1.0000
369	250	0.9719	0.6544	0.3857	1.0000
370	249	0.7452	0.4906	0.4003	1.0000
371	248	0.8273	0.4075	0.0155	1.0000
372	249	0.1651	0.6304	0.5967	1.0000
373	248	0.0164	0.1849	0.8656	1.0000

374	245	0.8566	0.6597	0.1591	1.0000
375	245	0.3527	0.8759	0.7146	1.0000
376	243	0.0117	0.5845	0.4260	1.0000
377	242	0.6893	0.7024	0.5525	1.0000
378	241	0.0600	0.5209	0.0466	1.0000
379	241	0.8724	0.4284	0.4977	1.0000
380	240	0.2682	0.8302	0.7746	1.0000
381	237	0.5942	0.8299	0.4719	1.0000
382	234	0.7868	0.0414	0.5342	1.0000
383	235	0.8051	0.7066	0.9393	1.0000
384	234	0.6568	0.1980	0.7251	1.0000
385	232	0.6863	0.6705	0.0386	1.0000
386	229	0.4385	0.8752	0.5121	1.0000
387	229	0.9110	0.0878	0.0338	1.0000
388	228	0.6229	0.9674	0.3864	1.0000
389	229	0.4993	0.0495	0.8167	1.0000
390	228	0.0473	0.3611	0.8597	1.0000
391	227	0.3941	0.6256	0.5121	1.0000
392	227	0.3425	0.5769	0.5387	1.0000
393	225	0.4709	0.2587	0.2967	1.0000
394	224	0.9736	0.1891	0.9090	1.0000
395	223	0.1664	0.7819	0.8312	1.0000
396	223	0.5675	0.8727	0.5063	1.0000
397	222	0.1806	0.1453	0.3661	1.0000
398	220	0.7105	0.4653	0.8076	1.0000
399	221	0.7182	0.5024	0.8621	1.0000
400	219	0.2334	0.4052	0.8605	1.0000
401	218	0.8076	0.7341	0.1392	1.0000
402	218	0.2933	0.8111	0.7306	1.0000
403	216	0.7503	0.1391	0.5513	1.0000
404	217	0.0020	0.1872	0.5585	1.0000
405	216	0.4252	0.1409	0.0540	1.0000
406	213	0.9789	0.1464	0.0685	1.0000
407	213	0.2367	0.6919	0.6014	1.0000
408	212	0.4845	0.8427	0.4863	1.0000
409	210	0.7473	0.3240	0.5013	1.0000
410	210	0.3331	0.6064	0.2818	1.0000
411	210	0.5214	0.3221	0.7285	1.0000
412	203	0.4690	0.3959	0.4581	1.0000
413	201	0.1821	0.7713	0.9723	1.0000
414	201	0.6648	0.4695	0.8862	1.0000
415	199	0.7292	0.6007	0.3925	1.0000
416	196	0.5321	0.3227	0.8654	1.0000
417	195	0.0590	0.0056	0.5145	1.0000
418	190	0.4174	0.7484	0.3328	1.0000

419	190	0.3447	0.6821	0.3139	1.0000
420	189	0.0295	0.3898	0.3912	1.0000
421	189	0.2450	0.3766	0.8112	1.0000
422	187	0.4374	0.4391	0.4368	1.0000
423	186	0.8255	0.6858	0.1245	1.0000
424	186	0.4684	0.6205	0.1976	1.0000
425	185	0.2194	0.7723	0.3071	1.0000
426	179	0.3006	0.1556	0.5719	1.0000
427	179	0.2625	0.5167	0.5010	1.0000
428	178	0.4167	0.1087	0.4232	1.0000
429	176	0.6257	0.6371	0.5302	1.0000
430	175	0.1134	0.6277	0.5568	1.0000
431	174	0.5429	0.2197	0.7682	1.0000
432	173	0.0440	0.3326	0.3518	1.0000
433	171	0.7457	0.7184	0.5160	1.0000
434	170	0.2217	0.3473	0.8526	1.0000
435	164	0.9289	0.8902	0.4845	1.0000
436	160	0.4136	0.3102	0.4252	1.0000
437	156	0.2519	0.2363	0.5420	1.0000
438	155	0.8410	0.9258	0.2255	1.0000
439	155	0.5494	0.3219	0.0112	1.0000
440	154	0.9380	0.6053	0.8753	1.0000
441	153	0.3819	0.1060	0.0006	1.0000
442	152	0.3586	0.8302	0.4858	1.0000
443	152	0.9746	0.8218	0.3553	1.0000
444	152	0.2013	0.2255	0.3945	1.0000
445	145	0.1868	0.0977	0.3986	1.0000
446	144	0.4834	0.9656	0.5275	1.0000
447	143	0.2922	0.1915	0.5397	1.0000
448	142	0.5439	0.4116	0.4538	1.0000
449	140	0.6726	0.2060	0.9502	1.0000
450	139	0.5064	0.3122	0.9098	1.0000
451	137	0.6378	0.6051	0.9954	1.0000
452	135	0.6258	0.5325	0.0009	1.0000
453	135	0.4828	0.1536	0.5135	1.0000
454	130	0.2334	0.9241	0.0085	1.0000
455	128	0.5566	0.6570	0.0765	1.0000
456	127	0.4700	0.4040	0.5144	1.0000
457	127	0.7197	0.2380	0.8629	1.0000
458	127	0.5014	0.4778	0.3754	1.0000
459	126	0.4762	0.1666	0.4762	1.0000
460	127	0.2445	0.7684	0.9611	1.0000
461	126	0.6803	0.5192	0.9884	1.0000
462	124	0.0504	0.2786	0.3795	1.0000
463	123	0.2139	0.2044	0.5976	1.0000

464	121	0.2996	0.6575	0.3577	1.0000
465	121	0.4809	0.2362	0.4706	1.0000
466	119	0.8107	0.3745	0.8778	1.0000
467	116	0.6103	0.6286	0.1255	1.0000
468	113	0.7325	0.3140	0.8689	1.0000
469	111	0.4445	0.1701	0.0121	1.0000
470	111	0.4089	0.2179	0.4645	1.0000
471	109	0.9049	0.7840	0.3351	1.0000
472	107	0.2798	0.0216	0.9451	1.0000
473	106	0.5429	0.2249	0.9259	1.0000
474	104	0.4336	0.4617	0.3773	1.0000
475	102	0.8283	0.9399	0.3601	1.0000
476	100	0.5598	0.7000	0.1023	1.0000
477	100	0.5643	0.2752	0.5611	1.0000
478	97	0.2412	0.7757	0.4637	1.0000
479	96	0.1338	0.8773	0.9983	1.0000
480	97	0.9035	0.0464	0.4003	1.0000
481	95	0.3552	0.4568	0.3437	1.0000
482	94	0.0365	0.6672	0.8897	1.0000
483	91	0.5961	0.6703	0.1859	1.0000
484	92	0.4044	0.3746	0.3483	1.0000
485	91	0.1782	0.8773	0.5939	1.0000
486	91	0.6875	0.7653	0.3146	1.0000
487	89	0.8559	0.9931	0.3770	1.0000
488	90	0.5772	0.1449	0.6777	1.0000
489	88	0.5495	0.2144	0.5714	1.0000
490	87	0.6491	0.7216	0.3394	1.0000
491	87	0.5325	0.1954	0.6265	1.0000
492	86	0.4359	0.1637	0.8582	1.0000
493	86	0.1365	0.3296	0.3878	1.0000
494	85	0.7006	0.8301	0.3358	1.0000
495	85	0.1120	0.3854	0.5750	1.0000
496	85	0.4383	0.2198	0.8840	1.0000
497	82	0.7813	0.3017	0.8749	1.0000
498	81	0.9342	0.4135	0.9338	1.0000
499	79	0.6301	0.2422	0.8409	1.0000
500	77	0.9050	0.2496	0.4427	1.0000
501	77	0.6963	0.7256	0.4766	1.0000
502	77	0.9503	0.0351	0.3984	1.0000
503	76	0.8250	0.8653	0.1988	1.0000
504	76	0.5362	0.2783	0.9390	1.0000
505	74	0.0715	0.6899	0.4022	1.0000
506	74	0.8104	0.1467	0.5468	1.0000
507	75	0.4835	0.2578	0.5661	1.0000
508	73	0.7533	0.7814	0.2121	1.0000
509	73	0.6038	0.3333	0.5467	1.0000
510	73	0.1163	0.1067	0.3756	1.0000
511	73	0.5778	0.3613	0.5728	1.0000
512	72	0.4532	0.6800	0.3651	1.0000
513	71	0.1229	0.3114	0.8882	1.0000
514	72	0.2355	0.3146	0.8987	1.0000
515	71	0.9552	0.4379	0.4899	1.0000
516	70	0.7004	0.7601	0.3785	1.0000
517	69	0.8263	0.1314	0.4153	1.0000
518	68	0.9709	0.7222	0.7873	1.0000
519	69	0.9497	0.5626	0.9165	1.0000
520	68	0.8225	0.9847	0.9641	1.0000
521	67	0.9627	0.9920	0.9024	1.0000
522	67	0.0854	0.8061	0.9656	1.0000
523	67	0.8930	0.5495	0.4680	1.0000
524	66	0.0047	0.5172	0.4958	1.0000
525	65	0.7066	0.8284	0.2613	1.0000
526	65	0.6950	0.0565	0.4529	1.0000
527	65	0.3137	0.8108	0.7981	1.0000
528	66	0.8426	0.4426	0.8849	1.0000
529	65	0.6553	0.2393	0.9134	1.0000
530	65	0.4841	0.1851	0.0971	1.0000
531	65	0.5776	0.7378	0.3452	1.0000
532	64	0.0469	0.0867	0.8003	1.0000
533	63	0.6270	0.4814	0.9190	1.0000
534	62	0.1699	0.9367	0.6406	1.0000
535	62	0.9365	0.4930	0.9273	1.0000
536	61	0.9378	0.5139	0.1385	1.0000
537	61	0.2650	0.7693	0.3445	1.0000
538	61	0.8753	0.1905	0.4328	1.0000
539	62	0.5564	0.1927	0.9636	1.0000
540	61	0.9063	0.0965	0.3319	1.0000
541	61	0.9913	0.7585	0.4739	1.0000
542	61	0.5167	0.2322	0.5206	1.0000
543	61	0.3251	0.7030	0.5476	1.0000
544	60	0.6675	0.8933	0.6254	1.0000
545	60	0.1720	0.3668	0.6474	1.0000
546	60	0.1588	0.4372	0.7947	1.0000
547	61	0.6397	0.6880	0.8905	1.0000
548	59	0.9055	0.1940	0.0114	1.0000
549	59	0.9842	0.6138	0.1417	1.0000
550	59	0.9868	0.0463	0.3170	1.0000
551	59	0.5124	0.7310	0.3361	1.0000
552	59	0.3652	0.3893	0.4142	1.0000
553	59	0.6514	0.7007	0.4624	1.0000

```
         554     59   0.8603   0.0016   0.7502   1.0000
         555     59   0.6405   0.2196   0.8760   1.0000
         556     58   0.3604   0.5934   0.0041   1.0000
         557     59   0.6224   0.2573   0.2855   1.0000
         558     59   0.9336   0.0363   0.3233   1.0000
         559     58   0.0195   0.9543   0.3852   1.0000
         560     59   0.5368   0.6991   0.4098   1.0000
         561     59   0.9690   0.7169   0.4986   1.0000
         562     58   0.6685   0.0488   0.5164   1.0000
         563     59   0.9522   0.4532   0.5250   1.0000
         564     58   0.0792   0.5303   0.5377   1.0000
         565     59   0.5886   0.1787   0.5597   1.0000
         566     59   0.1376   0.3407   0.5719   1.0000
         567     59   0.3914   0.6878   0.8200   1.0000
         568     58   0.1142   0.5531   0.8772   1.0000
         569     58   0.3761   0.6282   0.8753   1.0000
         570     58   0.2205   0.7165   0.9047   1.0000
         571     58   0.2196   0.7024   0.4282   1.0000
         572     57   0.7300   0.2101   0.5497   1.0000
         573     58   0.2458   0.4251   0.7518   1.0000
         574     58   0.2611   0.3337   0.8250   1.0000
------------------------------------------------------------------------------

  COPY OF FILE.CDR:
  ==== == =======

TITL ALFA1
CELL
20.846  20.909  27.057 102.40  95.33 119.62
LATTICE
P
SYMMETRY
X, Y, Z
CONTENT
CL  O   N   C  H
1  110 65  328  500
&CONTROL IHKL=1,DSFOU=0.90,NSET=25,NCYCLE=42,RVMIN=100.,
IATOMS=1,FROLW=-2.5,FSIGip=3.7,DELTLV=1.1,IFOUSG=1,
IBREAK=0,IEXCC=0,NITER=3,IPHASE=2,ITEST=1,EHLIM=1.0,
BINIT=0.6923080/


++++++++++++++++++++++++++++++++++++++++++++++++++++++++++++++
   Job finished on 19- 2-2024   at 19:36:22
   Elapsed time:        73 seconds
++++++++++++++++++++++++++++++++++++++++++++++++++++++++++++++
```

```
============================================================
   XLENS_SMARV241: A DELTA DIRECT-METHODS PROGRAM
   Crystal structure solution with the SMAR phasing algorithm
   Copyright Prof. Jordi Rius (Palleiro)
   Rius J. Acta Cryst (2020) A76 489-493
   Institut de Ciencia de Materials de Barcelona (CSIC)
   Date is 19- 2-2024   at 19:33:26
 ============================================================


     [pro-pro-phe-phe-gly-].MeOH.H20 in P2(1)2(1)2(1) 86 ATOMS UNITAT ASIM

    Crystal data:
     a =   13.999 =     alpha=   90.000
     b =   21.602 =     beta=    90.000
     c =   21.615 =     gamma=   90.000

     Reciprocal lattice constants (a, b, c):
     (sinth/l)2=  0.001276*H2 + 0.000536*K2 + 0.000535*L2 +
                 0.000000*HK + 0.000000*HL + 0.000000*KL

     Unit cell volume (a,b,c) =      6536.51

     Unit cell (a,b,c) is P centered
      nodes at:
       1)  0.000000       0.000000       0.000000

      Symmetry operations:
      R11  R12  R13     R21  R22  R23     R31  R32  R33    T1     T2     T3
  1)    1    0    0       0    1    0       0    0    1    0.00   0.00   0.00
  2)   -1    0    0       0   -1    0       0    0    1    0.50   0.00   0.50
  3)   -1    0    0       0    1    0       0    0   -1    0.00   0.50   0.50
  4)    1    0    0       0   -1    0       0    0   -1    0.50   0.50   0.00

          Unit cell contents
      Symbol  Atomic_number    Number in cell    type    scat_power
        O          8              56             1          8.00
        N          7              40             2          7.00
        C          6             248             3          6.00
        H          1             328             4          1.00
```

```
     X-RAYS

     RANDOM starting phases

--------------------------------------------------------------

     Intensity data information:
     ==========================

     Input data are F
     DSMIN (Angs.):            0.8909
     DSFOU (Angs.):            1.0000
--------------------------------------------------------------

     FORBIDDEN REFLECTIONS IN DATA FILE *.HKL:
     =========================================

    H    K    L        XO       DXO      NEQ
--------------------------------------------------------------

     Rsigma(F) of equivalent reflections
     Rsig:    0.00000  Nref:         0

     ds_interval        Rsigma       Nref

     N. UNIQUE REFLECTIONS (measured,theory):        3858        3859


     INFORMATION OF DATA IN P1:
     MEAS. INTENSITIES (OBS+UNOBS)IN XOHEM.HKL=        13695
     NOT MEASURED INTENSITIES in XOUNMEAS.HKL=           2
     FRACTION MEASURED INTENSITIES >DSFOU:     1.000
     HMAX=   13  KMAX=   21  LMAX=   21


--------------------------------------------------------------

     SCALING OF MEASURED INTENSITIES:
     ================================


      SCALE FACTOR:        0.8514  F(EXP)/SK = F(ABS)
      B OVERALL   :        5.1116
      R(Wilson)   :       64.4334
      DSFOU       :        1.0000
```

```
 FACDF       :        2.0000  Observed if XO > FACDF*sig(XO)

 SHELL  DMEAN  DINF  SFC2/SFO2  Nref  f.unobs
   1     2.15  1.71   0.72708    2746  0.0000
   2     1.49  1.36   1.49652    2710  0.0066
   3     1.26  1.19   1.16095    2753  0.0189
   4     1.13  1.08   0.92127    2707  0.0369
   5     1.04  1.00   0.85929    2779  0.0489

 -------------------------------------------------------------

DATA NORMALIZING:
================

NUMBER OF INPUT REFLEXIONS =            13695
F(ABS)  =  F(EXP) /          0.8514

              CENT    ACENT     HK0     0KL     H0L    REST
</E2-1/>      0.968   0.736   0.987   1.251   1.008   0.718

 E2MEAN      :    0.945229

<E**2> ACCORDING TO PARITY GROUPS ( D-SPACING > 1.000
   ALL     GGG     GGU     GUG     GUU     UGG     UGU     UUG     UUU
E^2   1.000   0.976   1.076   0.951   0.952   1.101   1.040   0.941   0.963
N     13695    1688    1711    1711    1712    1721    1716    1716    1720

--------------------------------------------------------------

PHASE REFINEMENT DETAILS:
========================
TOTAL N. OF E-VALUES =     13695
D-SPACING CUT-OFF =    1.00
<E>, <E2> =    0.8692  1.0000

N. SETS AND CYCLES =       25     28
ELIM FOR K REFLECTIONS =    0.00
N.REFLECTIONS > EKLIM =     13695
ELIM FOR H REFLECTIONS =    1.00
N.REFLECTIONS > EHLIM =      4760
N. ATOMS IN UNIT_CELL (NCELL)  =   344
PIXELS(0); P_ATOMS(1); OMIT(2) =     1
FRAC. RANDOMLY DELETED ATOMS = 0.000

GRID SPACING IN ANGS. =    0.33
```

N.GRID POINTS (XYZ) = 40 64 64
LOWER SDV FACTOR FOR MASK = -2.50
ESD FACT FOR PEAK ACCEPTANCE (ATOMS=1,2) = 4.00
ESD FACTOR IN SEARCH (DIF.FOURIER) = 1.10

ITER	SRO2	SDEL2	2S_RDM	P	Q	RDEL	%0	%-1	CCro’	CCro”
1	0.331E+10	0.581E+10	-0.0249	0.5204	0.8801	1.3755	49.856	0.183	0.0184	0.6015
2	0.332E+10	0.581E+10	-0.4946	0.5807	0.8664	0.9525	51.681	0.073	0.3486	0.6968
3	0.332E+10	0.581E+10	-0.5654	0.5856	0.8670	0.8872	52.474	0.051	0.3967	0.7322
4	0.332E+10	0.581E+10	-0.6030	0.5931	0.8652	0.8553	52.809	0.056	0.4209	0.7287
5	0.332E+10	0.581E+10	-0.5864	0.5926	0.8710	0.8772	52.769	0.057	0.4081	0.7267
6	0.332E+10	0.581E+10	-0.6333	0.5969	0.8765	0.8400	52.904	0.045	0.4378	0.7372
7	0.332E+10	0.581E+10	-0.6606	0.6019	0.8683	0.8095	52.975	0.056	0.4569	0.7328
8	0.332E+10	0.581E+10	-0.6752	0.6024	0.8759	0.8031	53.129	0.048	0.4648	0.7433
9	0.332E+10	0.581E+10	-0.6759	0.6042	0.8805	0.8088	52.991	0.046	0.4633	0.7287
10	0.332E+10	0.581E+10	-0.6860	0.6056	0.8778	0.7974	53.098	0.051	0.4704	0.7384
11	0.332E+10	0.581E+10	-0.7005	0.6090	0.8781	0.7866	53.187	0.054	0.4790	0.7362
12	0.332E+10	0.581E+10	-0.6869	0.6092	0.8793	0.8016	53.022	0.052	0.4693	0.7385
13	0.332E+10	0.581E+10	-0.6998	0.6120	0.8819	0.7941	53.236	0.049	0.4763	0.7301
14	0.332E+10	0.581E+10	-0.7121	0.6106	0.8753	0.7738	53.127	0.049	0.4870	0.7399
15	0.332E+10	0.581E+10	-0.7240	0.6142	0.8766	0.7668	53.260	0.051	0.4934	0.7513
16	0.332E+10	0.581E+10	-0.7240	0.6145	0.8861	0.7766	53.176	0.052	0.4906	0.7360
17	0.332E+10	0.581E+10	-0.7157	0.6130	0.8885	0.7858	53.099	0.049	0.4849	0.7414
18	0.332E+10	0.581E+10	-0.7341	0.6138	0.8814	0.7611	53.185	0.047	0.4990	0.7554
19	0.332E+10	0.581E+10	-0.7479	0.6187	0.8835	0.7543	53.333	0.054	0.5058	0.7380
20	0.332E+10	0.581E+10	-0.7581	0.6181	0.8802	0.7402	53.306	0.056	0.5139	0.7649
21	0.332E+10	0.581E+10	-0.7378	0.6152	0.8868	0.7641	53.058	0.052	0.4995	0.7425
22	0.332E+10	0.581E+10	-0.7473	0.6173	0.8842	0.7542	53.273	0.046	0.5058	0.7495
23	0.332E+10	0.581E+10	-0.7569	0.6215	0.8872	0.7518	53.235	0.063	0.5097	0.7468
24	0.332E+10	0.581E+10	-0.7792	0.6247	0.8925	0.7380	53.373	0.050	0.5218	0.7690
25	0.332E+10	0.581E+10	-0.8308	0.6377	0.9081	0.7150	53.690	0.054	0.5459	0.7930
26	0.332E+10	0.581E+10	-1.0404	0.6751	0.9198	0.5545	54.646	0.030	0.6601	0.8625
27	0.332E+10	0.581E+10	-1.1562	0.6987	0.9002	0.4427	55.499	0.013	0.7289	0.9023
28	0.332E+10	0.581E+10	-1.1689	0.7023	0.8987	0.4322	55.762	0.018	0.7356	0.9068

SUMMARY OF SOLUTIONS:

SET	CCH	SEED	CCK	NCYC	NITER	RVAL(ini,end)		PKS(ini,end)		RO/SIG
1	0.9364	0.9615380	0.8643	28	3	20.7	11.9	122	83	14.18

TOTAL NUMBER OF CYCLES IS 28 IEXCC = 0

PEAK SEARCH:
===========

SOLUTION N. 1 R = 11.890917 ITERATIONS= 3

NUMBER OF E AND CUT-OFF VALUE 2206 0.70
GRID: NX= 40 NY= 64 NZ= 64 SIZE(ANGS)= 0.33
ATOMS IN UNIT CELL: (SOUGHT) 344; (FOUND) 335
ATOMS IN ASYMMETRIC UNIT= 83
RO IN WFOURIER: MAX., SIGMA, MEAN: 0.31427E+05 0.33971E+04 0.81140E-05
MIN. INTERPEAK DISTANCE (A): 0.70

PEAK_NR	HEIGHT	X/A	Y/B	Z/C	MULT
1	1000	0.0278	0.2339	0.9987	1.0000
2	912	0.8246	0.7711	0.5771	1.0000
3	900	0.3895	0.7339	0.3281	1.0000
4	885	0.4233	0.1209	0.1208	1.0000
5	874	0.9126	0.9542	0.6415	1.0000
6	870	0.2488	0.0930	0.0153	1.0000
7	822	0.4566	0.3706	0.0633	1.0000
8	812	0.6927	0.6908	0.3652	1.0000
9	804	0.4937	0.1356	0.2372	1.0000
10	800	0.5781	0.9675	0.4041	1.0000
11	794	0.4239	0.0451	0.2652	1.0000
12	793	0.1790	0.9883	0.5605	1.0000
13	767	0.8715	0.6990	0.4248	1.0000
14	765	0.9050	0.6405	0.6710	1.0000
15	759	0.4594	0.3764	0.2265	1.0000
16	759	0.6004	0.9925	0.2338	1.0000
17	735	0.7010	0.2525	0.0157	1.0000
18	718	0.3758	0.1589	0.1551	1.0000
19	705	0.6437	0.2617	0.1251	1.0000
20	683	0.5027	0.5774	0.2414	1.0000
21	676	0.4318	0.3441	0.1238	1.0000
22	675	0.6788	0.1603	0.0754	1.0000
23	669	0.4985	0.3608	0.1763	1.0000
24	670	0.8505	0.6697	0.6224	1.0000
25	667	0.9631	0.5901	0.4552	1.0000
26	657	0.5596	0.9408	0.0725	1.0000
27	654	0.6034	0.4795	0.4089	1.0000
28	647	0.0963	0.4531	0.7292	1.0000
29	635	0.4726	0.9748	0.9903	1.0000
30	625	0.5836	0.9352	0.1379	1.0000
31	623	0.9044	0.1265	0.0888	1.0000
32	619	0.4003	0.1641	0.2256	1.0000
33	617	0.3990	0.2300	0.2475	1.0000
34	615	0.7460	0.7980	0.4849	1.0000
35	615	0.1801	0.9274	0.5453	1.0000

36	605	0.5430	0.9325	0.4419	1.0000
37	602	0.5921	0.9974	0.1710	1.0000
38	598	0.7061	0.1419	0.0129	1.0000
39	597	0.8861	0.7268	0.4795	1.0000
40	590	0.6545	0.4148	0.3921	1.0000
41	587	0.6514	0.3754	0.2249	1.0000
42	583	0.5195	0.4650	0.4531	1.0000
43	582	0.8172	0.9102	0.7677	1.0000
44	581	0.4499	0.8212	0.6139	1.0000
45	575	0.0052	0.6282	0.4201	1.0000
46	573	0.0973	0.8892	0.5576	1.0000
47	565	0.9974	0.0427	0.0048	1.0000
48	564	0.1973	0.3643	0.6853	1.0000
49	562	0.0744	0.4977	0.4654	1.0000
50	562	0.1087	0.7667	0.8180	1.0000
51	550	0.7642	0.4727	0.4528	1.0000
52	548	0.7483	0.3532	0.1380	1.0000
53	541	0.6464	0.9317	0.0313	1.0000
54	520	0.5295	0.7514	0.1494	1.0000
55	496	0.5442	0.2132	0.8404	1.0000
56	475	0.4908	0.2711	0.8588	1.0000
57	467	0.3262	0.3982	0.3650	1.0000
58	456	0.7774	0.7765	0.9039	1.0000
59	450	0.1852	0.7821	0.8452	1.0000
60	448	0.0346	0.4495	0.4190	1.0000
61	442	0.2772	0.9066	0.0581	1.0000
62	440	0.4728	0.5278	0.8612	1.0000
63	436	0.5098	0.1044	0.5653	1.0000
64	427	0.7505	0.3615	0.2032	1.0000
65	425	0.3200	0.6220	0.1236	1.0000
66	414	0.2705	0.6941	0.2009	1.0000
67	398	0.2191	0.5879	0.9215	1.0000
68	391	0.6163	0.7375	0.0574	1.0000
69	384	0.9690	0.3819	0.3357	1.0000
70	384	0.5402	0.7515	0.0842	1.0000
71	380	0.3104	0.6703	0.0796	1.0000
72	372	0.1547	0.3830	0.1868	1.0000
73	373	0.9721	0.3576	0.3928	1.0000
74	369	0.0328	0.3414	0.1285	1.0000
75	370	0.7497	0.2377	0.3438	1.0000
76	358	0.0873	0.3924	0.1433	1.0000
77	335	0.1949	0.7769	0.9119	1.0000
78	312	0.1989	0.0447	0.2100	1.0000
79	304	0.6569	0.5307	0.1164	1.0000
80	294	0.8851	0.7865	0.2919	1.0000
81	245	0.3251	0.9010	0.7301	1.0000
82	158	0.8522	0.5486	0.1095	1.0000
83	139	0.5089	0.5445	0.1921	1.0000

```
  COPY OF FILE.CDR:
  ==== == =======

[pro-pro-phe-phe-gly-].MeOH.H20 in P2(1)2(1)2(1) 86 ATOMS UNITAT ASIM
CELL
13.999  21.602  21.615  90.  90.  90.
LATTICE
P
SYMMETRY
X, Y, Z
-X+1/2, -Y, Z+1/2
-X, Y+1/2, -Z+1/2
 X+1/2, -Y+1/2, -Z
CONTENT
  O   N   C   H
 56  40 248 328
```

```
&CONTROL IHKL=0,DSFOU=1.0,NSET=25,RVMIN=100.,NCYCLE=28,
IATOMS=1,FROLW=-2.5,FSIGip=4.0,DELTLV=1.1,BINIT=0.9615380,
NITER=3,IFOUSG=1,IBREAK=0,IPHASE=2,iexcc=0,itest=1,EHLIM=1.0/

++++++++++++++++++++++++++++++++++++++++++++++++++++++++++++++
   Job finished on 19- 2-2024   at 19:33:49
   Elapsed time:        23 seconds
++++++++++++++++++++++++++++++++++++++++++++++++++++++++++++++
```

```
  XLENS_SMARV241: A DELTA DIRECT-METHODS PROGRAM
  Crystal structure solution with the SMAR phasing algorithm
  Copyright Prof. Jordi Rius (Palleiro)
  Rius J. Acta Cryst (2020) A76 489-493
  Institut de Ciencia de Materials de Barcelona (CSIC)
  Date is 19- 2-2024   at 19:32:28
============================================================


    TITL SUCROSE OCTA-ACETATE AT -100C

   Crystal data:
    a =   18.350 =     alpha=   90.000
    b =   21.441 =     beta=    90.000
    c =    8.350 =     gamma=   90.000

    Reciprocal lattice constants (a, b, c):
    (sinth/l)2=  0.000742*H2 + 0.000544*K2 + 0.003586*L2 +
                0.000000*HK + 0.000000*HL + 0.000000*KL

    Unit cell volume (a,b,c) =      3285.24

    Unit cell (a,b,c) is P centered
     nodes at:
      1)  0.000000       0.000000       0.000000

     Symmetry operations:
     R11  R12  R13     R21  R22  R23     R31  R32  R33    T1     T2     T3
 1)    1    0    0       0    1    0       0    0    1    0.00   0.00   0.00
 2)   -1    0    0       0   -1    0       0    0    1    0.50   0.00   0.50
 3)    1    0    0       0   -1    0       0    0   -1    0.50   0.50   0.00
 4)   -1    0    0       0    1    0       0    0   -1    0.00   0.50   0.50

         Unit cell contents
     Symbol  Atomic_number    Number in cell    type    scat_power
       O           8              76            1          8.00
       C           6             112            2          6.00
       H           1             152            3          1.00

    X-RAYS
```

```
     RANDOM starting phases

--------------------------------------------------------------

     Intensity data information:
     ==========================

     Input data are F
     DSMIN (Angs.):            0.8392
     DSFOU (Angs.):            1.0000
--------------------------------------------------------------

     FORBIDDEN REFLECTIONS IN DATA FILE *.HKL:
     =========================================

    H    K    L        XO       DXO      NEQ
--------------------------------------------------------------

     Rsigma(F) of equivalent reflections
     Rsig:    0.00000  Nref:         0

     ds_interval        Rsigma       Nref

     N. UNIQUE REFLECTIONS (measured,theory):        1886        2026


     INFORMATION OF DATA IN P1:
     MEAS. INTENSITIES (OBS+UNOBS)IN XOHEM.HKL=         6437
     NOT MEASURED INTENSITIES in XOUNMEAS.HKL=         472
     FRACTION MEASURED INTENSITIES >DSFOU:     0.932
     HMAX=   18  KMAX=   21  LMAX=    8


--------------------------------------------------------------

     SCALING OF MEASURED INTENSITIES:
     ================================


      SCALE FACTOR:        0.8622  F(EXP)/SK = F(ABS)
      B OVERALL   :        1.8120
      R(Wilson)   :       37.3275
      DSFOU       :        1.0000
      FACDF       :        2.0000  Observed if XO > FACDF*sig(XO)
```

```
      SHELL  DMEAN  DINF  SFC2/SFO2  Nref  f.unobs
        1     2.15  1.71   0.79286    1256  0.0207
        2     1.49  1.36   1.32369    1260  0.0460
        3     1.26  1.19   1.18218    1254  0.0877
        4     1.13  1.08   0.87047    1331  0.0969
        5     1.04  1.00   0.92593    1336  0.1287

 -------------------------------------------------------------

     DATA NORMALIZING:
     ================

     NUMBER OF INPUT REFLEXIONS =              6437
     F(ABS)  =  F(EXP) /          0.8622

                   CENT    ACENT     HK0     0KL     H0L    REST
     </E2-1/>      0.968   0.736   1.051   1.192   1.048   0.741

      E2MEAN      :    0.998890

     <E**2> ACCORDING TO PARITY GROUPS ( D-SPACING > 1.000
        ALL     GGG     GGU     GUG     GUU     UGG     UGU     UUG     UUU
E^2   1.000   1.037   0.966   1.052   0.923   0.972   1.111   1.012   0.933
N      6437     809     816     809     828     815     780     788     792

--------------------------------------------------------------

     PHASE REFINEMENT DETAILS:
     ========================
    TOTAL N. OF E-VALUES =      6437
    D-SPACING CUT-OFF =    1.00
    <E>, <E2> =    0.8662  1.0000

    N. SETS AND CYCLES =       25     27
    ELIM FOR K REFLECTIONS =    0.00
    N.REFLECTIONS > EKLIM =      6437
    ELIM FOR H REFLECTIONS =    1.00
    N.REFLECTIONS > EHLIM =      2258
    N. ATOMS IN UNIT_CELL (NCELL)  =   188
    PIXELS(0); P_ATOMS(1); OMIT(2) =     1
    FRAC. RANDOMLY DELETED ATOMS = 0.000

    GRID SPACING IN ANGS. =    0.33
    N.GRID POINTS (XYZ) =     60    64    24
    LOWER SDV FACTOR FOR MASK =   -2.50
```

ESD FACT FOR PEAK ACCEPTANCE (ATOMS=1,2) = 3.75
ESD FACTOR IN SEARCH (DIF.FOURIER) = 1.50

ITER	SRO2	SDEL2	2S_RDM	P	Q	RDEL	%0	%-1	CCro'	CCro"
1	0.883E+09	0.158E+10	-0.0328	0.5169	0.8931	1.3772	49.707	0.173	0.0241	0.6020
2	0.887E+09	0.158E+10	-0.5061	0.5798	0.8893	0.9630	52.044	0.078	0.3524	0.7295
3	0.887E+09	0.158E+10	-0.5570	0.5817	0.8854	0.9101	52.597	0.040	0.3881	0.7225
4	0.887E+09	0.158E+10	-0.5971	0.5894	0.8790	0.8713	52.854	0.027	0.4148	0.7321
5	0.887E+09	0.158E+10	-0.6104	0.5933	0.8893	0.8721	52.829	0.054	0.4202	0.7349
6	0.887E+09	0.158E+10	-0.6331	0.5953	0.8957	0.8580	52.862	0.037	0.4335	0.7262
7	0.887E+09	0.158E+10	-0.6928	0.6073	0.8801	0.7947	53.432	0.039	0.4738	0.7146
8	0.887E+09	0.158E+10	-0.6729	0.6039	0.8849	0.8159	52.964	0.049	0.4603	0.7212
9	0.887E+09	0.158E+10	-0.6808	0.6037	0.8926	0.8156	52.866	0.036	0.4637	0.7443
10	0.887E+09	0.158E+10	-0.6986	0.6078	0.8823	0.7915	53.003	0.042	0.4770	0.7468
11	0.887E+09	0.158E+10	-0.7223	0.6112	0.8860	0.7749	53.394	0.038	0.4908	0.7307
12	0.887E+09	0.158E+10	-0.7275	0.6108	0.8854	0.7687	53.124	0.038	0.4946	0.7689
13	0.887E+09	0.158E+10	-0.7466	0.6169	0.8901	0.7603	53.620	0.040	0.5038	0.7530
14	0.887E+09	0.158E+10	-0.7372	0.6099	0.8925	0.7652	53.333	0.027	0.4996	0.7623
15	0.887E+09	0.158E+10	-0.7588	0.6145	0.8894	0.7451	53.448	0.034	0.5132	0.7467
16	0.887E+09	0.158E+10	-0.7700	0.6142	0.8962	0.7405	53.496	0.024	0.5189	0.7594
17	0.887E+09	0.158E+10	-0.7683	0.6150	0.8948	0.7415	53.459	0.024	0.5179	0.7769
18	0.887E+09	0.158E+10	-0.7520	0.6156	0.8944	0.7581	53.491	0.029	0.5067	0.7568
19	0.887E+09	0.158E+10	-0.7507	0.6164	0.8938	0.7596	53.666	0.029	0.5057	0.7568
20	0.887E+09	0.158E+10	-0.7573	0.6176	0.8987	0.7590	53.579	0.022	0.5082	0.7429
21	0.887E+09	0.158E+10	-0.7884	0.6233	0.8983	0.7332	53.732	0.027	0.5268	0.7681
22	0.887E+09	0.158E+10	-0.7756	0.6201	0.8928	0.7373	53.627	0.018	0.5212	0.7683
23	0.887E+09	0.158E+10	-0.8007	0.6250	0.9085	0.7328	53.723	0.036	0.5313	0.7720
24	0.887E+09	0.158E+10	-0.8194	0.6286	0.9173	0.7265	53.860	0.020	0.5395	0.7887
25	0.887E+09	0.158E+10	-0.9930	0.6597	0.9353	0.6019	54.872	0.009	0.6321	0.8416
26	0.887E+09	0.158E+10	-1.1634	0.6980	0.9074	0.4421	56.446	0.004	0.7309	0.9107
27	0.887E+09	0.158E+10	-1.1795	0.7016	0.9004	0.4224	56.444	0.005	0.7420	0.9173

SUMMARY OF SOLUTIONS:

SET	CCH	SEED	CCK	NCYC	NITER	RVAL(ini,end)		PKS(ini,end)		RO/SIG
1	0.9447	0.6923080	0.8834	27	5	12.8	5.7	68	47	11.25

TOTAL NUMBER OF CYCLES IS 27 IEXCC = 0

PEAK SEARCH:
===========

SOLUTION N. 1 R = 5.693436 ITERATIONS= 5

NUMBER OF E AND CUT-OFF VALUE 1066 0.70
GRID: NX= 60 NY= 64 NZ= 24 SIZE(ANGS)= 0.33
ATOMS IN UNIT CELL: (SOUGHT) 188; (FOUND) 188
ATOMS IN ASYMMETRIC UNIT= 47
RO IN WFOURIER: MAX., SIGMA, MEAN: 0.21268E+05 0.22923E+04 0.14914E-05
MIN. INTERPEAK DISTANCE (A): 0.70

PEAK_NR	HEIGHT	X/A	Y/B	Z/C	MULT
1	1000	0.8993	0.1556	0.6173	1.0000
2	980	0.3519	0.2690	0.2034	1.0000
3	969	0.2812	0.2074	0.4546	1.0000
4	945	0.3150	0.6906	0.0065	1.0000
5	908	0.6636	0.8206	0.5808	1.0000
6	884	0.9907	0.1765	0.2465	1.0000
7	874	0.2213	0.4210	0.3707	1.0000
8	862	0.4461	0.4573	0.2617	1.0000
9	822	0.4773	0.2559	0.1431	1.0000
10	803	0.8788	0.6521	0.4729	1.0000
11	796	0.0564	0.8461	0.4738	1.0000
12	780	0.8824	0.8011	0.2171	1.0000
13	776	0.1225	0.8237	0.1031	1.0000
14	741	0.2666	0.5166	0.0794	1.0000
15	682	0.6519	0.8898	0.1642	1.0000
16	677	0.7604	0.2599	0.1157	1.0000
17	676	0.4409	0.5613	0.2932	1.0000
18	671	0.6787	0.4089	0.1621	1.0000
19	662	0.7515	0.8150	0.0900	1.0000
20	652	0.4039	0.2467	0.0901	1.0000
21	645	0.5323	0.8190	0.5818	1.0000
22	633	0.3934	0.0606	0.9972	1.0000
23	631	0.8497	0.0117	0.1258	1.0000
24	619	0.9912	0.5736	0.2893	1.0000
25	608	0.7025	0.0284	0.5402	1.0000
26	604	0.1014	0.7229	0.4259	1.0000
27	592	0.8748	0.2194	0.6317	1.0000
28	591	0.0199	0.2175	0.9853	1.0000
29	588	0.8796	0.8052	0.0769	1.0000
30	585	0.3174	0.1627	0.5426	1.0000
31	585	0.8832	0.0472	0.6291	1.0000
32	583	0.6928	0.7689	0.0316	1.0000
33	581	0.2901	0.6589	0.4209	1.0000
34	571	0.9814	0.6208	0.2084	1.0000
35	564	0.3906	0.1749	0.0865	1.0000
36	554	0.9428	0.4077	0.0676	1.0000
37	554	0.7312	0.7953	0.5878	1.0000
38	549	0.7214	0.8823	0.0716	1.0000
39	531	0.1717	0.1061	0.9839	1.0000
40	520	0.7857	0.5835	0.3789	1.0000
41	514	0.5584	0.1941	0.4565	1.0000
42	499	0.2165	0.8994	0.0421	1.0000
43	497	0.4231	0.8241	0.3921	1.0000
44	469	0.9402	0.6260	0.0552	1.0000
45	467	0.5353	0.5168	0.1344	1.0000
46	455	0.4721	0.5190	0.2324	1.0000
47	444	0.5031	0.5736	0.6867	1.0000

COPY OF FILE.CDR:
==== == =======

```
TITL SUCROSE OCTA-ACETATE AT -100C
CELL
18.350  21.441  8.350  90.  90.  90.
LATTICE
P
SYMMETRY
X,Y,Z
```

```
1/2-X, -Y, 1/2+Z
1/2+X, 1/2-Y, -Z
-X, 1/2+Y, 1/2-Z
CONTENT
O   C   H
76  112  152
&CONTROL IHKL=0,DSFOU=1.0,NSET=25,RVMIN=100.,NCYCLE=27,
IATOMS=1,FROLW=-2.5,FSIGIP=3.75,DELTLV=1.5,BINIT=0.6923080,
NITER=5,IFOUSG=1,IBREAK=0,IPHASE=2,IEXCC=0,EHLIM=1.0,
ITEST=1/
++++++++++++++++++++++++++++++++++++++++++++++++++++++++++++++
   Job finished on 19- 2-2024   at 19:32:41
   Elapsed time:        13 seconds
++++++++++++++++++++++++++++++++++++++++++++++++++++++++++++++
```
